# Genetic Pathway in Acquisition and Loss of Vancomycin Resistance in a Methicillin Resistant *Staphylococcus aureus* (MRSA) Strain of Clonal Type USA300

**DOI:** 10.1371/journal.ppat.1002505

**Published:** 2012-02-02

**Authors:** Susana Gardete, Choonkeun Kim, Boris M. Hartmann, Michael Mwangi, Christelle M. Roux, Paul M. Dunman, Henry F. Chambers, Alexander Tomasz

**Affiliations:** 1 Laboratory of Microbiology, The Rockefeller University, New York, New York, United States of America; 2 Molecular Genetics Laboratory, Instituto de Tecnologia Química e Biológica da Universidade Nova de Lisboa, Oeiras, Portugal; 3 Department of Neurology, Mount Sinai School of Medicine, New York, New York, United States of America; 4 Department of Microbiology and Immunology, University of Rochester Medical Center, Rochester, New York, United States of America; 5 Division of Infectious Diseases, Department of Medicine, University of California San Francisco, San Francisco, California, United States of America; Children's Hospital Boston, United States of America

## Abstract

An isolate of the methicillin-resistant *Staphylococcus aureus* (MRSA) clone USA300 with reduced susceptibility to vancomycin (SG-R) (i.e, vancomycin-intermediate *S. aureus*, VISA) and its susceptible “parental” strain (SG-S) were recovered from a patient at the end and at the beginning of an unsuccessful vancomycin therapy. The VISA phenotype was unstable *in vitro* generating a susceptible revertant strain (SG-rev). The availability of these 3 isogenic strains allowed us to explore genetic correlates of antibiotic resistance as it emerged *in vivo*. Compared to the susceptible isolate, both the VISA and revertant strains carried the same point mutations in *yycH*, *vraG*, *yvqF* and *lspA* genes and a substantial deletion within an intergenic region. The revertant strain carried a single additional frameshift mutation in *vraS* which is part of two component regulatory system VraSR. VISA isolate SG-R showed complex alterations in phenotype: decreased susceptibility to other antibiotics, slow autolysis, abnormal cell division and increased thickness of cell wall. There was also altered expression of 239 genes including down-regulation of major virulence determinants. All phenotypic properties and gene expression profile returned to parental levels in the revertant strain. Introduction of wild type *yvqF* on a multicopy plasmid into the VISA strain caused loss of resistance along with loss of all the associated phenotypic changes. Introduction of the wild type *vraSR* into the revertant strain caused recovery of VISA type resistance. The *yvqF/vraSR* operon seems to function as an on/off switch: mutation in *yvqF* in strain SG-R turns on the *vraSR* system, which leads to increase in vancomycin resistance and down-regulation of virulence determinants. Mutation in *vraS* in the revertant strain turns off this regulatory system accompanied by loss of resistance and normal expression of virulence genes. Down-regulation of virulence genes may provide VISA strains with a “stealth” strategy to evade detection by the host immune system.

## Author Summary

The extensive use of antibiotics has led to the selection of methicillin-resistant *S. aureus* (MRSA) strains that are resistant to most antimicrobial agents and a treatment of choice against such strains is vancomycin. However, during the last decade reports of treatment failure with vancomycin non-susceptible MRSA (e.g., vancomycin intermediate *S. aureus*, VISA) began to appear in the clinical setting. In this paper we analyze the mechanism of resistance in a VISA strain that belongs to the epidemic and highly virulent MRSA clone USA300. We had 3 isogenic isolates available for analysis: the vancomycin susceptible parental strain recovered from the patient before the onset of therapy; the VISA strain recovered at the time of clinical treatment failure and a susceptible revertant of the VISA strain acquired during in vitro passage. We identified genetic differences among the three strains through whole genome sequencing. In this strain, the key genetic change responsible for vancomycin resistance was in the functionally connected *yvqF/vraSR* - two component sensory regulatory system involved with the control of cell wall metabolism of the bacteria. The same genetic change also caused repression of virulence related properties which may help the resistant bacteria to evade the host immune system.

## Introduction

The extensive use of antibiotics in the clinical environment has led to the appearance of a wide variety of drug resistance mechanisms among all bacterial pathogens, including *S. aureus*. While most of these resistance mechanisms are associated with the acquisition of “foreign” genetic elements – others involve a more direct selection of bacterial mutants that can survive and overcome the inhibitory effects of the antibiotic. Examples for such adaptive resistance mechanism are provided by the appearance of *S. aureus* strains with decreased susceptibility to the antibiotic vancomycin. These so-called VISA isolates (for Vancomycin intermediate *S. aureus*) were usually recovered from patients with vancomycin treatment failure (for review see [1]). Although development of VISA type resistance has been described during vancomycin treatment of methicillin susceptible *S. aureus* (MSSA) infections [2], the vast majority of VISA isolates were identified in MRSA strains, i.e., multidrug resistant isolates of *S. aureus* against which the therapy of choice has been vancomycin for some time. This highly concentrated selective pressure on MRSA is undoubtedly the primary reason for the emergence of vancomycin resistant mutants in the most frequent lineages of these already multiresistant strains. Thus, isolates exhibiting VISA type resistance have been identified in strains belonging to most of the major epidemic MRSA clones. The first VISA isolate – MU50 described in Japan in 1997 [3] and VISA isolates from New Jersey, Michigan [4] and from Portchester [5] all share sequence type ST5 of the “New York/Japan” MRSA clone. Similarly, a series of VISA isolates exhibiting gradually decreasing levels of susceptibility to vancomycin belong to ST105 which is a single locus variant of the same ST5 clone [6]–[8]. Several VISA isolates were identified in the ST239 Brazilian MRSA [9] and a recently described VISA isolate – VISA-BRAGA – belongs to the EMRSA-15 clone of ST22 [10]. Two most recent communications described recovery of isolates with VISA type resistance from infections caused by the USA300 clone belonging to ST8 [11]–[12]. The appearance of VISA type resistance in the genetic background of this highly virulent MRSA lineage – frequently associated with both community and hospital acquired infections – is of concern.

An intriguing feature of the VISA phenotype is the large number of physiological and morphological abnormalities that has been observed in most if not all of such bacteria. These abnormalities include changes in morphology, pattern of cell division, decrease in proneness to autolysis; suppression of hemolysis, abnormal structure of the cell surface, decreased virulence in animal models. The multiplicity of these phenotypic changes suggests that the mechanism of VISA-type vancomycin resistance involves alteration in (a) genetic determinant(s) that control such complex phenomena.

In this communication we describe microbiological, transcriptional and genetic analysis of the VISA type resistance to vancomycin in a clinical isolate belonging to the USA300 MRSA clone [11]. The results suggest that a mutated *yvqF* causing over-expression of the VraSR regulon is the key factor leading to the increase in the vancomycin MIC value and – more importantly – to the down-regulation of numerous virulence genes which may equip the bacteria with a strategy to evade immune surveillance during invasion and prolong survival in the host and also contribute to the unique clinical features of VISA type infections.

## Results

### Multiple and reversible alterations in the phenotype of a vancomycin resistant (VISA) isolate of the MRSA clone USA300

#### Decreased susceptibility to several cell wall inhibitors

**Figure 1:**
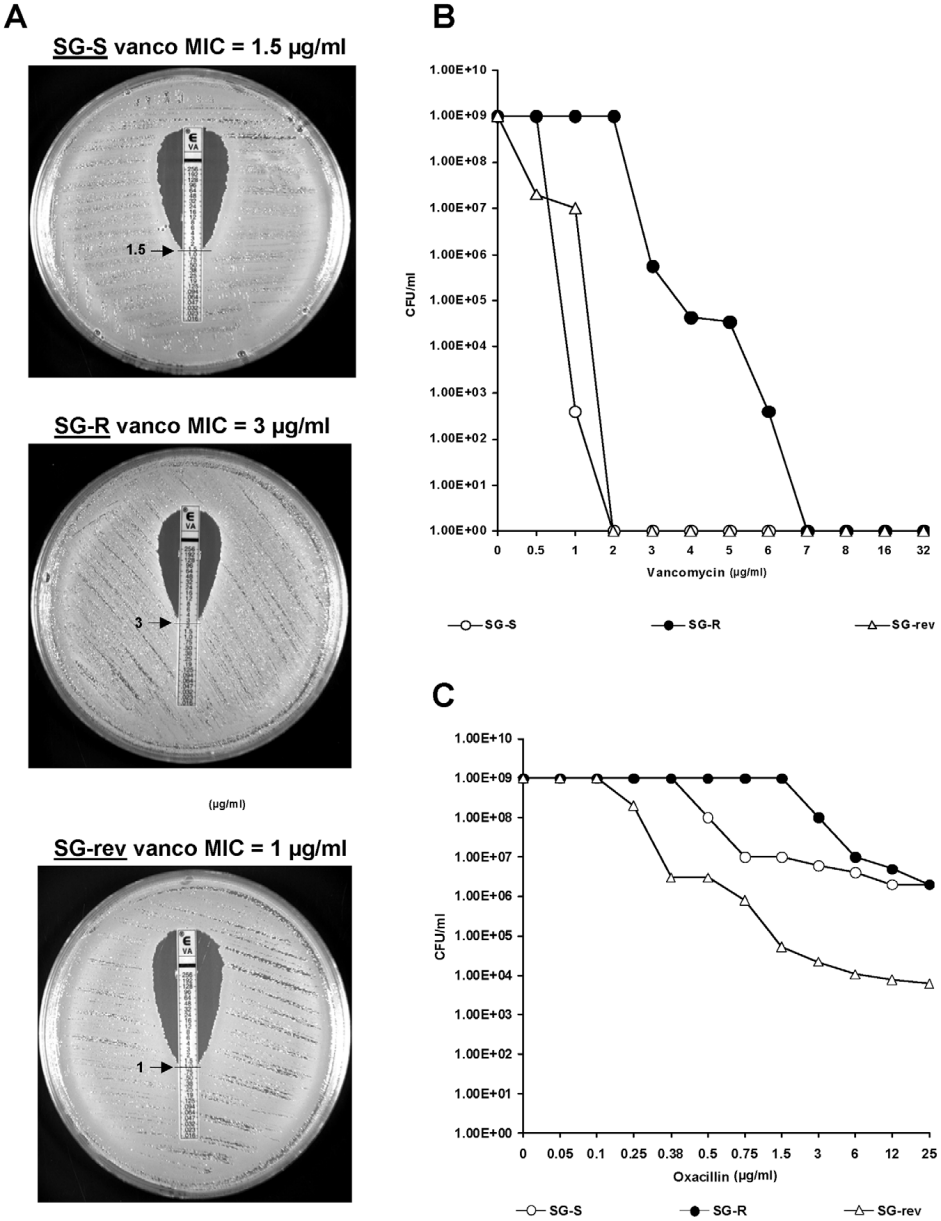
Vancomycin and oxacillin susceptibility profiles of strains SG-S, SG-R and SG-rev. Strains SG-S, SG-R and SG-rev were grown overnight in TSB and were used to determine vancomycin MIC values using the E-test with bacteria plated on TSA (**A**). Vancomycin and oxacillin susceptibility were also determined by population analysis (**B**) and (**C**).

The antibiotic susceptibility profile of the resistant isolate SG-R (San Francisco General Hospital-Resistant) recovered at the end of an unsuccessful vancomycin therapy was determined by the E-test and population analysis profile and was compared to the corresponding values of the “parental” strain SG-S (San Francisco General Hospital-Susceptible). The vancomycin MIC of SG-R was 3 µg/ml as compared to the MIC of 1 to 1.5 µg/ml in SG-S (Figure 1A). Plating the bacterial strains for population analysis showed that cultures of the resistant isolate SG-R were heterogeneous: the majority of cells could grow at 2 µg/ml with a 3 log_10_ decrease at 3 µg/ml, but subpopulations with higher MIC values for vancomycin were also present with low frequencies. In contrast, cultures of the parental strain SG-S and the revertant SG-rev were more homogeneous with no growth observable at 2 µg/ml (Figure 1B).

The resistant isolate SG-R also showed modest but significant increase in the MIC value for oxacillin from 0.5 to 3 µg/ml as shown by a shift in the population analysis profile– (see Figure 1C). The resistant isolate SG-R also showed increased MICs for daptomycin (from 0.25 to 1.5 µg/ml), fosfomycin (from 1.5 to 8 µg/ml) and moenomycin (from 0.6 to 1.25 µg/ml) (Table 1). There was no difference detectable between strains SG-R and SG-S in susceptibility to levofloxacin, rifampin, chloramphenicol, tetracycline and mupirocin (data not shown) and the three strains were equally susceptible to linezolid and tigecycline – two antibiotics frequently used in the therapeutic setting.

**Table 1:** Relevant phenotypic properties of the strains used in this study.

Strain	Van^a^	Oxa^a, b^	DP^a^	FM^a^	MO^a^	LZ	TGC	Autolysis rate	Cell Division	Thickness of the cell wall
SG-S	1–1.5	0.5	0.25	1.5	0.6	S	S	Normal	Normal	Normal
SG-R	3	3	1.5	8	1.25	S	S	Reduced	Defective	Thick
SG-rev	0.5–1	0.38	0.19	0.38	0.07	S	S	Normal	Normal	Normal

Van – vancomycin; Oxa – oxacillin; DP – daptomycin, FM – fosfomycin, MO – moenomycin, LZ – linezolid, and TGC - tigecycline. (a) MIC values in µg/ml. (b) strain USA300 is a heterogeneously resistant MRSA strain in which – similarly to other heteroresistant MRSA – the majority of cells expressed only marginal level of resistance to oxacillin [69]. Complete population analysis profiles of vancomycin and oxacillin resistance for the SG strains are shown in Figs 1 B and C, respectively.

In the isolate SG-rev (San Francisco General Hospital-revertant) that lost resistance to vancomycin during *in vitro* passage, the MIC values for various cell wall inhibitors were similar to or lower than the MICs for the “parental” strain (see Table 1).

PFGE of SmaI digested DNA confirmed that the three strains SG-S, SG-R and SG-rev had identical band patterns (data not shown).

#### Defective cell division and abnormal morphology

**Figure 2:**
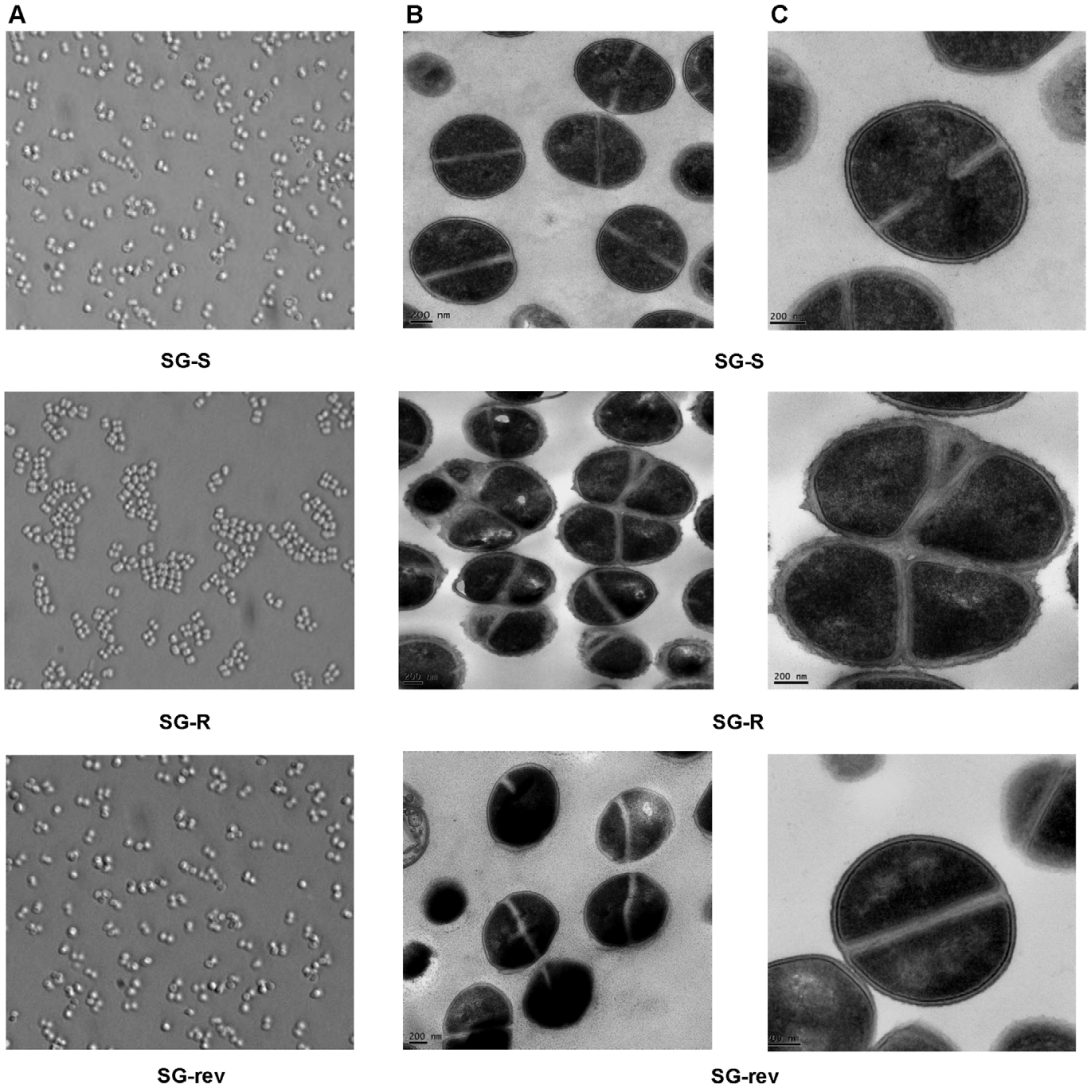
Phenotypes of strains SG-S, SG-R and SG-rev. Phenotypes of strains SG-S, SG-R and SG-rev were characterized by phase-contrast microscopy (**A**), and electron microscopy of thin sections (**B & C**).

Isolates SG-S, SG-R and SG-rev were grown in a nonselective medium under identical growth conditions and subjected to morphometric analysis by phase contrast and transmission electron microscopy. In contrast to the regularly shaped and well separated cells of the susceptible parental (SG-S) and revertant (SG-rev) cultures, the resistant strain SG-R grew in multicellular aggregates (>90% of cells), showed irregularly spaced and “thicker” than normal septae and cell walls surrounded by amorphous extracellular material (100% of the cells) (see figures 2A–C).

#### Reduced rate of Triton X-100 induced autolysis

**Figure 3:**
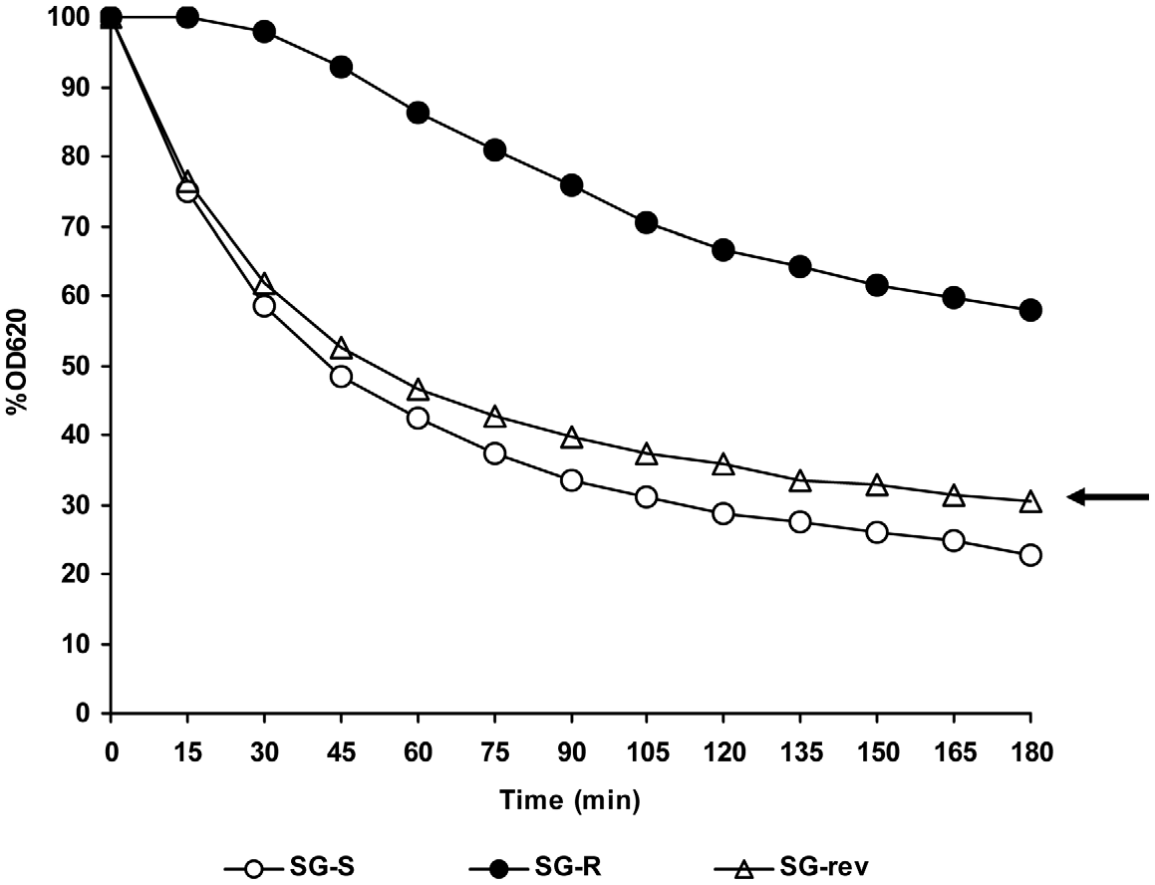
Triton X-100-stimulated autolysis of the SG-S, SG-R, and SG-rev strains. Cultures were suspended in Triton X-100 autolysis buffer to an initial OD_620_ of approximately 1.0, and the rates of autolysis were monitored as decrease of OD_620_ in time.

Liquid cultures of strains SG-R, SG-S and the revertant SG-rev grew with comparable rates in TSB in the logarithmic phase of growth. However, following back dilution of the overnight cultures, the resistant strain SG-R showed a considerable lag before resumption of growth, which occurred with the same rate as the parental and revertant strain (data not shown). The three strains were subjected to Triton X-100 stimulated autolysis and decrease in OD_620_ was followed every 15 minutes. The rate of autolysis of the resistant mutant SG-R was slower than that of the susceptible strain SG-S or the revertant SG-rev (Figure 3).

Peptidoglycan isolated from strains SG-S, SG-R and SG-rev were analyzed by reverse-phase HPLC. The muropeptide elution profiles of the three strains were virtually identical with one exception: the representation of the minor muropeptide component 1 – the monomeric disaccharide pentapeptide with no oligoglycine branches [13] – which was slightly increased in strain SG-R (data not shown).

Zymographic analyses [8] detected no significant differences between the autolytic enzyme profiles of the three strains (data not shown).

### Altered gene expression in the vancomycin resistant isolate SG-R

The transcriptional profile of the resistant isolate SG-R was compared to that of the susceptible parental strain SG-S and the revertant mutant SG-rev. Genes that showed a fold change cut off ≥2 accompanied by a p-value cut off ≤0.05 in at least one of the comparisons were selected for further investigation. All genes with significant altered expression in the three strains are listed in Table 2, Table S1 and Figure S1 and are categorized according to their functional group.

**Table 2:** Number of genes with significant transcriptional changes and respective functions in SG-R and SG-rev derived by Microarrays analysis.

	SG-R/SG-S			SG-R/SG-rev		
Functional Category	Total of genes with altered transcription	Number of genes Up-regulated	Number of genes Down-regulated	Total of genes with altered transcription	Number of genes Up-regulated	Number of genes Down-regulated
Ribosomal proteins: Synthesis and modification	17	—	17	17	—	17
Protein synthesis: Translation factors, and tRNA and rRNA base modification	3	—	3	3	—	3
Transport and binding proteins	20	8	12	18	6	12
Protein fate: protein and peptide secretion and trafficking/degradation of proteins, peptides, and glycopeptides	6	4	2	6	4	2
Post-translational modification, protein turnover and chaperones	2	2	—	2	2	—
Amino acid transport and metabolism/Amino acid biosynthesis	4	—	4	4	—	4
Energy metabolism	15	6	9	15	6	9
Pathogenesis	16	2	14	16	2	14
Regulatory functions	12	4	8	12	4	8
Cell envelope/Cell envelope biosynthesis	15	7	8	15	7	8
Biosynthesis of cofactors, prosthetic groups, and carriers: Menaquinone and ubiquinone	7	—	7	7	—	7
Inorganic ion transport and metabolism	6	—	6	6	—	6
Central intermediary metabolism	7	1	6	7	1	6
Biosynthesis of secondary metabolites	2	1	1	2	1	1
Signal transduction	7	2	5	7	2	5
DNA metabolism: DNA replication, recombination, and repair	4	1	3	4	1	3
Nucleotide transport and metabolism	2	—	2	2	—	2
Carbohydrate transport and metabolism	3	—	3	3	—	3
Fatty acid and phospholipid metabolism:	5	—	5	5	—	5
Detoxification	1	—	1	1	—	1
Drug resistance	2	1	1	2	1	1
Mobile and extrachromosomal element functions: Prophage functions	1	1	—	1	1	—
Unknown function	82	25	57	81	25	56
**Total of genes**	239	65	174	236	63	173

In the VISA strain SG-R 65 genes were up-regulated and 174 genes down-regulated – as compared to the parental strain SG-S (Tables 2, Table S1 and Figure S1). Out of these differentially transcribed genes, 20 encode for transport and binding proteins, 17 are linked to ribosomal processes, 16 to pathogenesis, 15 to energy metabolism, another 15 to cell envelope structure or biosynthesis, 12 to regulatory functions and 7 to central intermediary metabolism (Table 2). The majority of genes represented in Table S1 and Figure S1 (a total of 82 genes) are predicted to encode for proteins with unidentified functions. The altered transcription of a select group of these genes (*lytM*, *agrA*, *sgtB*, *orf2302*, *prsA*, *SceD*) was confirmed by Northern blot analysis (data not shown).

In the revertant isolate SG-rev the majority of genes that showed altered transcription in SG-R have returned to a pattern of normal transcription seen in the parental strain: only 8 genes with statistical significance (p≤0.05) could still be found differentially expressed in the comparison between the revertant strain and the parental strain – and among these genes was *vraG* (Table S1 and Figure S1) that is mutated in both SG-R and SG-rev (see Table 3).

**Table 3:** Mutations identified by whole genome sequencing analysis of SG-S, SG-R, and SG-rev strains.

			Locus (gene identification number)						
Number of mutation	Mutation	Effect of mutation	USA300 TCH1516	N315	Other	Function	SG-S	SG-R	SG-rev
1	C^27978^→A^27978^	A^165^→D^165^ (non - synonymous)	USA300HOU_0020	SA0019	***yycH***	Membrane protein. Part of the *yycFG* operon [14]	−	+	+
2	G ^737170^→A^737170^	G^551^→E^551^ (non - synonymous)	USA300HOU_0683	SA0617	***vraG***	ABC - transporter permease [16]	−	+	+
3	G ^1206012^→T^1206012^	D^109^→Y^109^ (non-synonymous)	USA300HOU_1133	SA1039	***lspA***	lipoprotein-specific type II signal peptidase [32], [70]	−	+	+
4	T^2027986^→C^2027986^	Y^220^→C^220^ (non - synonymous)	USA300HOU_1882	SA1702	***yvqF***	Conserved hypothetical protein. Part of the *vraSR* operon [22]	−	+	+
5	Deletion T^2027914^	Frameshift mutation. Premature stop codon at 234 aa	USA300HOU_1881	SA1701	***vraS***	Two-component sensor histidine kinase [21]	−	−	+
6	Deletion from T^1036471^ to A^1036838^	Deletion	2888 pb at the 5′ side – USA300HOU_0983 (*com k* – competence gene) 4409 pb at the 3′ side – USA300HOU_0984 (glycosyltransferase *GT1_gtfA_homolog*)			Intergenic region	−	+	+

### Genetic basis of antibiotic resistance

Whole genome sequencing analysis allowed identification of genetic differences among the three strains. Four nucleotide substitutions and a significant deletion of an intergenic region (367 bp) were found in the genome of the resistant isolate SG-R when compared to that of the parental strain SG-S (see Table 3). Each of the four mutations located in the *yycH* [14]–[15], *vraG* [16], *yvqF* [7], [17] and *lspA* [18] led to non-synonymous amino acid substitutions – in YycH (from non-polar to polar), in the ABC transporter permease VraG (from non-polar to polar), in YvqF (from polar to non-polar), and in LspA (from polar to polar). Detailed analysis of the deleted intergenic region showed that this fragment of DNA corresponds to the non-protein-coding (npc)RNA *Sau-02* identified by Abu-Qatouseh *et al.* in a study with small colony variants of *S. aureus* [19].

The revertant strain SG-rev carried the same mutations identified in SG-R but had an additional genetic alteration: a single nucleotide deletion in *vraS*. This mutation caused a frameshift in *vraS* and – presumably – a premature termination (at amino acid 234) of the histidine kinase VraS in the two-component system VraSR [20]–[21]. Sequencing confirmed each of the mutations present in the genomes of SG-R and SG-rev.

### Role of *yvqF* and *vraSR* in the expression of vancomycin resistance in strains SG-R and SG-rev

Whole genome sequencing provided evidence that the laboratory strain SG-rev may have lost the vancomycin resistant phenotype due to the deletion of a single nucleotide in the gene vraS suggesting the production of a “shortened” VraS histidine kinase. In S. aureus, the two genes yvqF and vraS were shown to be parts of the same operon [22], but a functional connection between the two genes was based most often on evidence provided by homology to determinants in other bacterial species: *liaF* (ortholog of *yvqF*) and *liaR* (ortholog of *vraR*) described in *B. subtilis*. The *yvqF* homologue *liaF* was shown to be a negative regulator of *liaR* [23]. Homologs of *yvqF* with similar function were also identified in *Streptococcus mutans* [24] and *Listeria monocytogenes* [25]. Independent evidence for a direct interaction between YvqF and VraS was provided by recent studies with the bacterial two-hybrid system [26].

These observations suggested that the mutated *yvqF* (and not the other 4 mutations present in SG-R) may represent the key genetic determinant associated with the acquisition of vancomycin resistance in strain SG-R. To test this hypothesis complementation experiments were performed.

### Complementation experiments

#### Abolishment of vancomycin resistance in strain SG-R – by over-expression of wild-type *yvqF*

**Figure 4:**
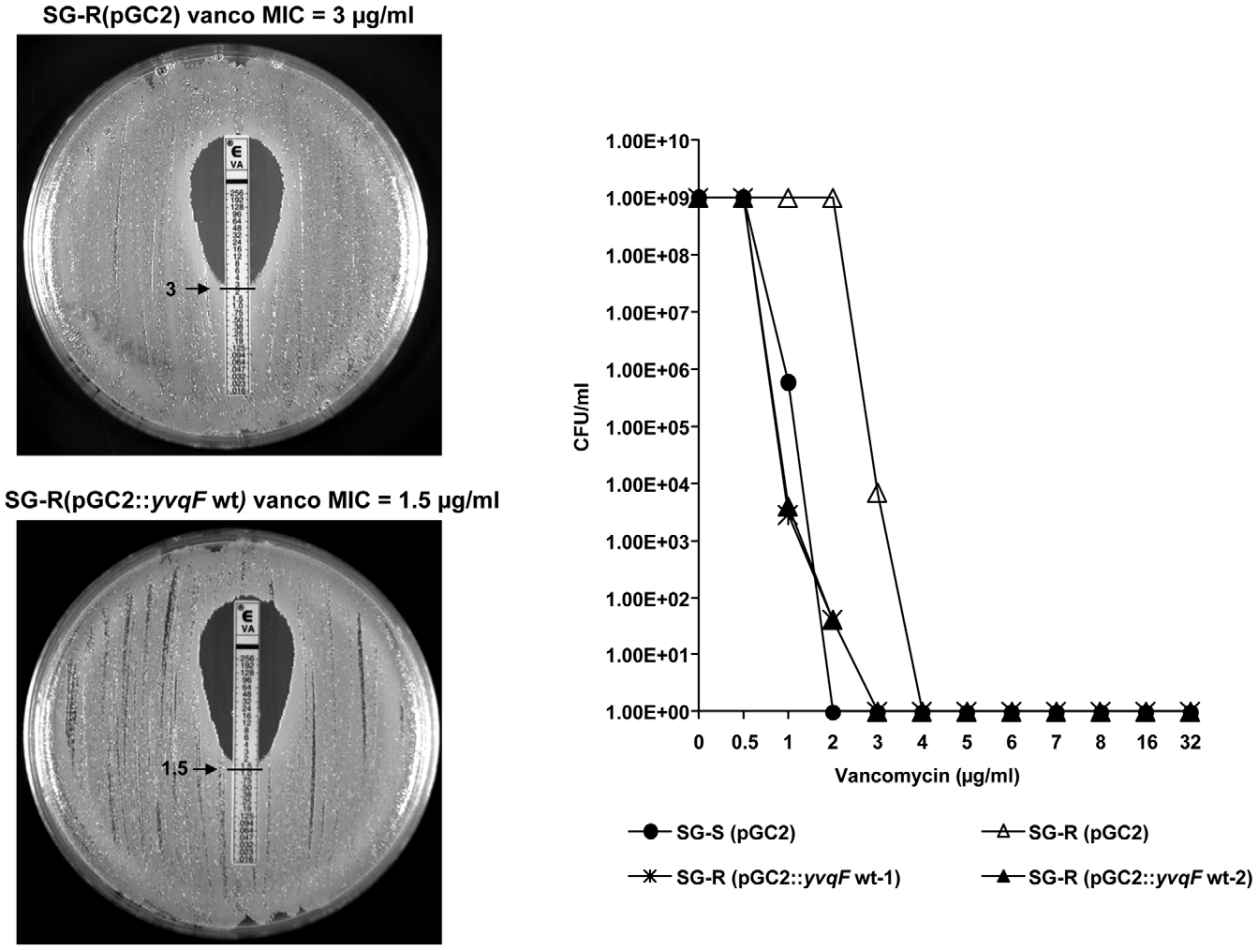
Loss of vancomycin resistance in strain SG-R complemented with the wild type *yvqF* gene. Vancomycin susceptibilities were evaluated by the E-test and by population analysis – as described in the Methods.

The wild type form of *yvqF* linked to the main promoter region of the *vraSR* operon [22] was fused to the plasmid pGC2 and introduced into strain SG-R. Over-expression of pGC2 carrying the wild type form of *yvqF* gene in strain SG-R completely abolished the vancomycin resistant phenotype: MIC was reduced from 3 to 1.5 µg/ml (Figure 4).

The complemented strain also recovered normal cell division, normal rate of TritonX-100 induced autolysis and low oxacillin MIC value – all characteristics of the parental strain SG-S (data not shown).

#### Recovery of vancomycin resistance in strain SG-rev by over-expression of wild type *vraSR*

**Figure 5:**
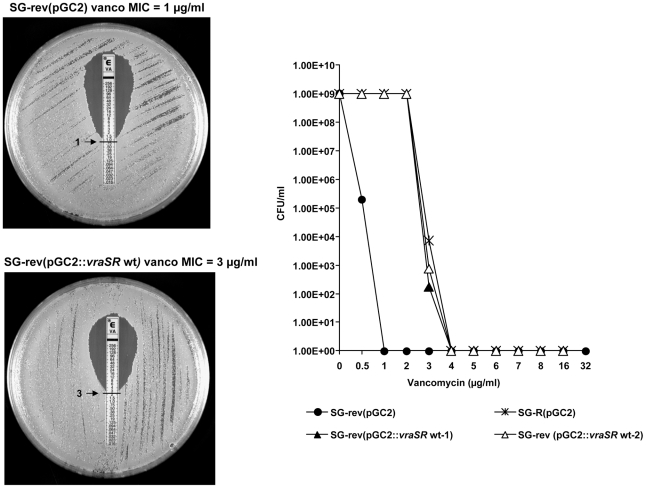
Recovery of vancomycin resistance in strain SG-rev complemented with the wild type *vraSR* genes. Vancomycin susceptibilities were evaluated by the E-test and by population analysis – as described in the Methods.

The wild type form of the *vraSR* genes linked to the promoter region of the *vraSR* operon was fused to pGC2 and introduced into strain SG-rev. Over-expression of the wild type form of *vraSR* in SG-rev caused full recovery of VISA type vancomycin resistance: MIC was increased from 1 to 3 µg/ml (Figure 5).

### Role of *yvqF* in the transcription of *vraSR*: evidence for *yvqF* as a negative regulator

**Figure 6:**
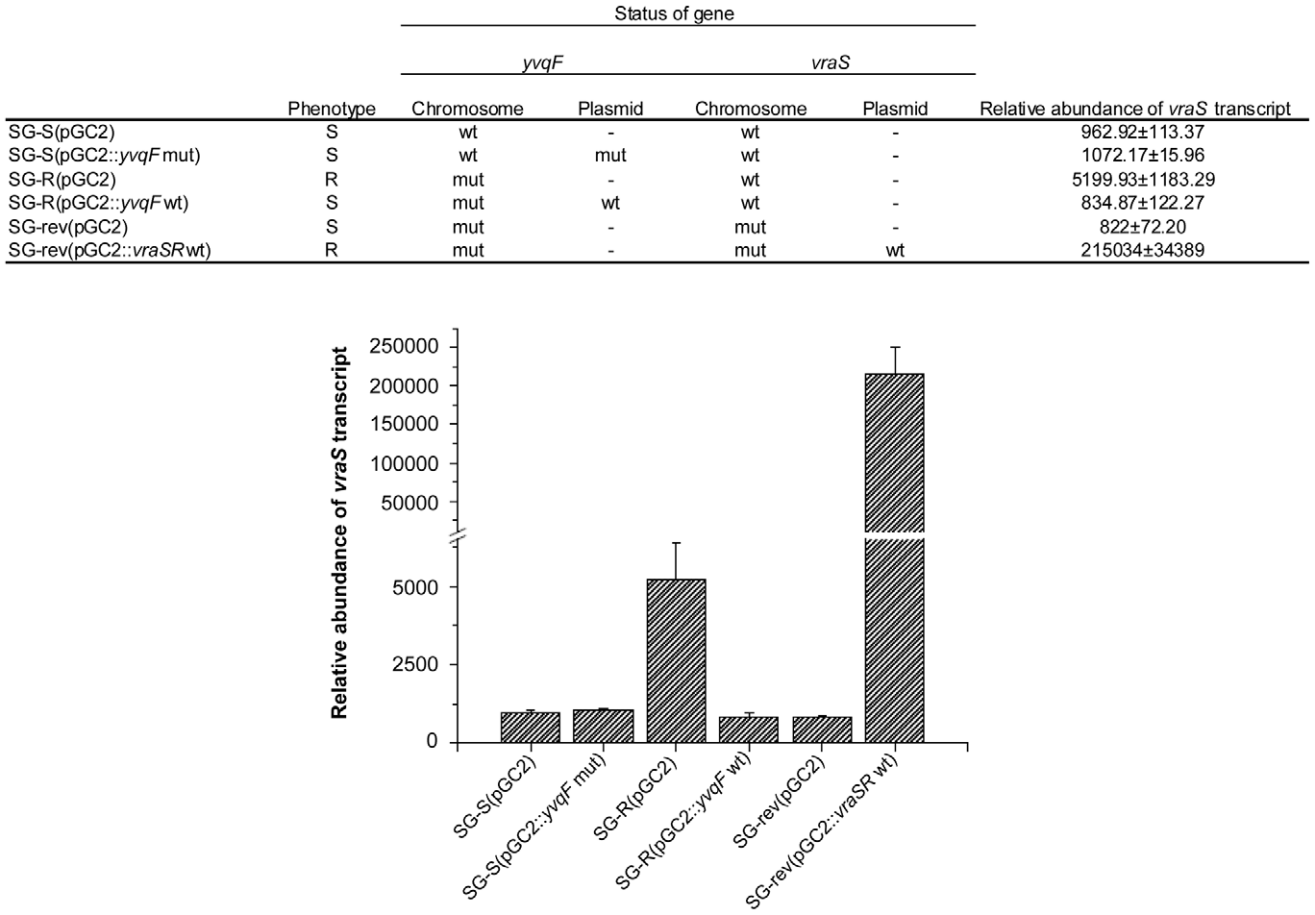
Transcription of *vraS* in strains SG-S, SG-R and SG-rev and their various genetic constructs. The expression of *vraS* mRNA was determined by qRT-PCR. As internal control, the expression was also determined in each sample for the *pta* mRNA.

In order to further explore the connection between *yvqF* and *vraSR* at the transcriptional level, qRT-PCR was performed. The total RNA from strains SG-S(pGC2::*yvqF*mut), SG-R(pGC2::*yvqF*wt) and SG-rev(pGC2::*vraSR*wt) was converted into cDNA and the amount of *vraS* cDNA expressed by each mutant was determined by qRT-PCR. Strains SG-S, SG-R and SG-rev with the carrier plasmid pGC2 were used as controls. As shown in Figure 6 transcription of *vraS* was up-regulated in the resistant strain SG-R (five-fold compared to the SG-S strain) and highly up-regulated in SG-rev(pGC2::*vraSR*wt) (approximately 220 fold compared to the parental vancomycin susceptible strain), i.e., in two strains that carried a mutated form of YvqF. The huge increase of *vraS* transcript in SG-rev(pGC2::*vraSR*wt) was presumably caused by the high copy number of the plasmid carrying the *vraS* gene in addition to the chromosomal copy of *vraS.*

Interestingly, SG-S(pGC2::*yvqF*mut) and SG-R(pGC2::*yvqF*wt) that were both vancomycin susceptible and carried both wild type and mutated forms of the *yvqF* gene exhibited the same level of *vraS* transcription as the parental strain SG-S that carried the chromosomal copy of wild type *yvqF* gene (see Figure 6). This indicates that decrease of *vraS* transcription in SG-R(pGC2::*yvqF*wt) is not due to the high copy number of the introduced plasmid that carried the wild type *yvqF* gene but rather it was due to the functional activity of wild type YvqF.

These observations identify the wild type form of YvqF as a negative controller of *vraSR* preventing up-regulation of this genetic system under normal conditions of growth. SG-rev containing the mutated form of *yvqF* did not show over-expression of *vraS* presumably due to the mutation in the *vraS* gene which caused loss of the histidine kinase activity of VraS and shutdown of the VraSR system.

### Widespread role of YvqF in the control of gene expression

Further evidence that YvqF is part of the regulatory circuits of *S. aureus* was provided by additional qRT-PCR assays. Transcription of *prsA*, *sgtB*, *orf1* in the *vraSR* operon, and *orf* SA2221 - all belonging to the VraSR regulon [21] – were over expressed in the VISA strain SG-R, but the transcription of these four genes became down-regulated in SG-R(pGC2::*yvqF*wt) (see Figure S2-A). The same pattern was also evident for *lytM* that appears to be an additional member of the VraSR regulon (data not shown).

Genes involved in different functional categories such as transport and binding proteins (*sptC*), protein fate (*sspB*), fatty acid and phospholipid metabolism (*acpD*), inorganic ion transport and metabolism (*sirA*), detoxification (*sodM*), and energy metabolism (*lctE and narK*) were found to be over-expressed in the VISA mutant complemented with the wild type form of *yvqF* (see Figure S2-B).

### Down-regulation of virulence related genes in the VISA strain SG-R

**Figure 7:**
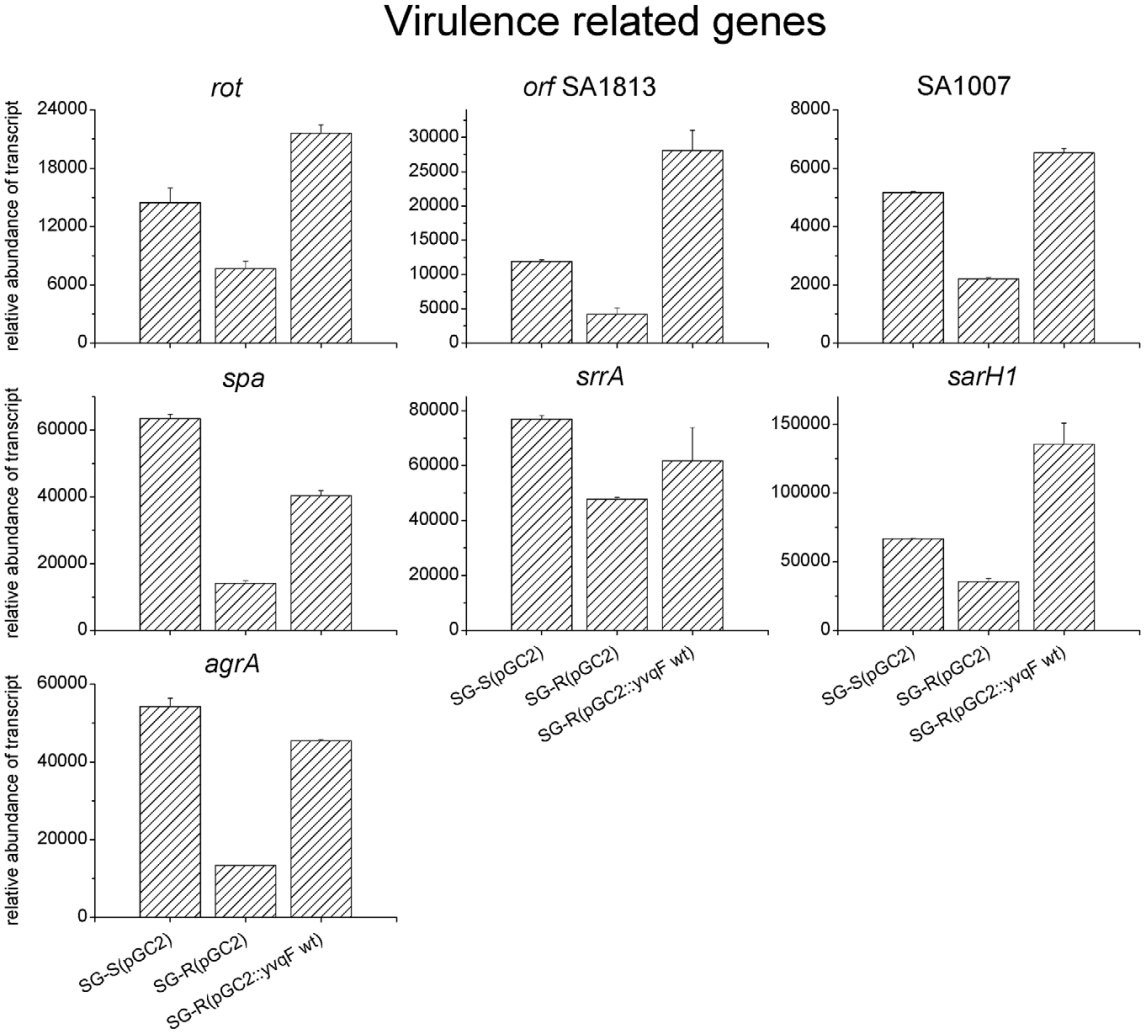
Transcription of virulence related genes in strains SG-S(pGC2), SG-R(pGC2) and SG-R(pGC2::*yvqF* wt). The expression of each mRNA was determined by qRT-PCR. As internal control, the expression was also determined in each sample for the *pta* mRNA. *orf* SA1007 encodes for alpha-hemolysin, and *orf* SA1813 encodes for an hypothetical protein, similar to leukocidin chain LukM.

Interestingly, a direct or indirect transcriptional connection between *yvqF* and important virulence related genes such as *agrA*, *rot*, *sarH1*, *spa*, alpha-hemolysin, etc, was also observed. All these genes were down-regulated in the VISA strain SG-R when compared with their transcription in the parental strain SG-S and their inhibition of transcription was reversed in SG-R(pGC2::*yvqF*wt) in parallel with the loss of resistance to vancomycin (see Figure 7).

## Discussion

During the extensive three weeks long vancomycin chemotherapy the invading USA300 strain SG-S acquired mutations in four genes that were identified in the isogenic strain SG-R in the determinants *yvqF, yycH, vraG* and *lspA*. An additional alteration was also detected in the resistant bacterium in an intergenic region.

### Primary role of the *yvqF* mutation in the VISA type resistance of strain SG-R

**Gene** ***yvqF*** is part of the VraSR operon [22] and it encodes for a membrane protein the biological function of which is not fully understood. Mutations in *yvqF* were found in several VISA strains including strain JH2, one of the “early” VISA isolates in the JH series of strains which belong to the MRSA clone ST-5 [7] and a variety of other VISA isolates from diverse geographic sources [7], [17]. A single substitution of tyrosine to cysteine in the intracellular domain of YvqF in SG-R caused over-expression of the *vraS* transcript (see Figure 6) suggesting that the intact YvqF acts as an inhibitor of VraS function and the mutation has completely abolished this inhibitory function. One may speculate that introduction of a cysteine residue may cause conformational change of YvqF by forming intra or inter-molecular disulfide bond. The results of the complementation experiments shown in Figures 4 and 6 clearly identify the mutation in this gene as the genetic change responsible for the increased vancomycin MIC value of strain SG-R and also for the other pleiomorphic alterations identified in the resistant bacterium (e.g., thick cell wall, slow autolysis, abnormal cell separation, etc).

### YvqF is a negative regulator of the *vraSR* operon

Besides identifying the mutation in *yvqF* as the genetic change responsible for the increased vancomycin MIC value, the complementation experiments also settle a more general issue related to the functioning of the VraSR system. The role of YvqF as a negative regulator was based until now on homology with orthologs of this gene in other bacterial species. The *S. aureus* YvqF shows amino acid similarity to LiaF of *B. subtilis* that was shown to be a potent negative regulator of the expression of LiaR which is the ortholog of *S. aureus* VraR [23]. Deletion analysis of the *liaIHGFSR* operon revealed that LiaF is an essential component of the LiaRS signaling system for maintaining it in a repressed state. Additional evidence for the role of LiaF as a negative controller of LiaSR was also provided by studies in *Streptococcus mutans* and *Listeria monocytogenes* [24]–[25].

The results of the complementation experiments described in this communication together with the titration of the *vraS* transcript provide a direct experimental evidence for the unequivocal identification of YvqF as an inhibitor of VraS function in *S. aureus* and this interpretation is in accord with the proposed role of YvqF in other gram positive bacteria [23]–[25]. Evidence for a direct interaction between YvqF and VraS was also supported by McCallum et al. who used the bacterial two-hybrid system to demonstrate such an interaction [26]. However, the findings with the two-hybrid system were interpreted as evidence for a role of YvqF as an activator – rather than as a repressor – of signal transduction. Further experimental work will be needed to sort out these different interpretations.

**Gene** ***yycH*** is part of an operon containing the structural determinants of the two-component system WalKR (YycFG) that controls part of the autolytic system of *S. aureus* and biofilm formation as well [14]–[15]. It was shown that the YycI and YycH orthologs in *B. subtilis* interact to negatively control the YycF activity [27]. Substitution of alanine to aspartic acid in the YycH protein of strain SG-R appears to be in the extracellular domain of the protein. The mutation may affect the inhibitory activity of YycH on the YycG sensor domain. It was previously reported that integration of an IS*256* in the promoter region of the *yycFGHI* operon led to over-expression of the *yycFGHI* genes and VISA type vancomycin resistance in a clinical isolate recovered from a tracheal secretion [28]–[29].

Whether or not the mutation in the *yycH* gene identified in strain SG-R contributes to the phenotype of this VISA strain is unclear. The slight difference in autolytic rate observed between SG-S and SG-rev (see arrow in Figure 3) and the minor compositional abnormality detected in the SG-R peptidoglycan (slight decrease in the representation of muropeptide 1) may represent consequences of this mutation in the WalKR system [14].

**Gene** ***vraG*** encodes an ABC transporter permease which is part of an ATP-binding cassette the expression of which is controlled by yet another two-component regulatory system GraSR [16]. GraSR was shown to be a major contributor to the VISA type resistance in some clonal types of *S. aureus* [30]–[31]. Deletion of *vraG* in the VISA strain Mu50 caused hypersensitivity to vancomycin [16]. The recently sequenced JH9 clinical isolate was also shown to carry a truncated YycH protein and an amino acid substitution in VraG [7]. It was proposed that the VraFG ABC transporter may enhance export of cell wall/teichoic acid precursors [16]. The particular point mutation in *vraG* of strain SG-R may or may not contribute to the phenotype of the resistant strain. However, the transcript of *vraG* remains highly expressed in both SG-R and SG-rev indicating that transcription of this gene is not under the control of VraSR system.

**Gene** ***lspA*** – a lipoprotein-specific type II signal peptidase – has not been seen in mutated form in other VISA isolates. The *lspA* gene was shown to be a virulence factor in *S. aureus* [18]. The mutation of aspartic acid to tyrosine identified in strain SG-R should cause loss of catalytic activity of this protein since the aspartic acid residue is one of two catalytic amino acids in the active site [32]. The mutation in *lspA* should cause loss or decrease of virulence in strain SG-R.

**Intergenic region Sau-02** – a small non-protein-coding RNA – was first described in a study focusing on the identification of sRNAs that were differentially expressed in small colony variants (SCVs) of *S. aureus*. In SCVs *Sau-02* was found to be down-regulated in the stationary phase of growth [19] and it was suggested that small regulatory RNAs may play a role in the control of growth phase in SCVs of *S. aureus*. Deletion of *Sau-02* from the SG-R chromosome could be a possible contributor to the aberrant growth pattern identified in strain SG-R.

### Genetic mechanism of the loss of antibiotic resistance in the revertant strain SG-rev

The USA300 VISA strain SG-R was shown to be unstable in the absence of drug selection and the revertant strain SG-rev has almost fully recovered (except for 8 genes) the transcriptional pattern of the parental strain. In addition – in SG-rev – each of the abnormalities observed in the VISA strain such as slow autolysis and abnormalities of cell wall structure – have returned to the normal properties characteristic of the parental strain. The antibiotic MIC values of the revertant isolate have also decreased “back” to the MIC values of the parental strain SG-S or even slightly below those values.

Full genome sequencing of the revertant strain showed that these bacteria have retained each of the five genetic alterations identified in the resistant strain but gained a single additional frameshift mutation in *vraS* which appears to be responsible for reversion of all the phenotypic and transcriptional changes detected in the resistant isolate.

The single nucleotide deletion in the *vraS* gene should lead to the production of a truncated histidine kinase VraS. The two-component system VraSR is a key regulator of the cell wall stimulon of *S. aureus* and it seems to act as a sentinel, sensing perturbations in cell wall synthesis and activating the transcription of several genes to preserve and repair the compromised cell wall [21], [33]. It was previously shown that in mutants with inactivated *vraSR* the level of vancomycin and oxacillin resistance was reduced [21], [34]. Moreover, mutations in *vraSR* were also identified in several clinical VISA isolates [7], [35], [36]. Most interestingly, the same VraS mutation detected in the SG-rev strain was also identified in a recently identified revertant strain JH9_rev_ (manuscript in preparation) and in a susceptible derivative of the VISA strain MU50 [30]. These observations strongly suggest that in several *S. aureus* lineages, a common mechanism leading to the loss of vancomycin resistance involves a mutation in the *vraS* gene. Such a hypothesis is corroborated by the results of the complementation experiment: the construct SG-rev(pGC2::*vraSR*wt) showed full recovery of vancomycin resistance and over-expression of *vraSR* (Figures 5 and 6).

The powerful impact of *vraS* mutation on the transcription of a wide range of genetic determinants is indicated by the finding that in the revertant strain SG-rev the great majority of the 239 genes which were shown to have altered transcription in the resistant mutant SG-R returned to the normal pattern of transcription as seen in the parental strain SG-S.

### Altered transcription of genes in the antibiotic resistant VISA isolate SG-R

In total, 239 genes showed altered transcription in the resistant strain compared to the parental strain SG-S. All but eight of these transcriptional changes were fully reversed in the revertant strain SG-rev. The genes with altered transcription included signal transduction and regulatory functions.

Out of the 191 genes whose expression is commonly altered when *S. aureus* is challenged with inhibitory concentrations of cell wall inhibitors [33], 50 genetic determinants involved in cell wall metabolism (*murZ*, *sgtB*), stress responses (*msr*, *htrA*, and *psrA*) and other cellular functions were found to be differentially transcribed in the resistant mutant. Among these 50 genes, 29 were previously classified as members of the cell wall stimulon of *S. aureus* [33].

Interestingly, 17 genes involved in the synthesis and modification of ribosomal proteins and 2 translation initiation factors were also down-regulated in the resistant strain. One can speculate that a delay in the translation process may be a strategy used by the resistant bacteria to compensate their imbalanced metabolic state due to the significant alterations in gene transcription.

It is noteworthy that 28 of the 46 members of the VraSR regulon [21], including the *vraSR* operon (*orf1, yvqF, vraS* and *vraR*), were found up-regulated in the resistant strain SG-R. Among all the transcriptional studies carried out with several different VISA isolates [20], [37]–[41], the transcriptional profile of SG-R resembled most closely that of the VISA isolate JH9 which also carries a mutation in the *yvqF* gene and exhibits over-expression of the VraSR operon and regulon [7], [39] (see Table S1).

### Down-regulation of virulence-related genes in the VISA isolate SG-R: a “stealth” strategy of invasion?

The vancomycin susceptible parental isolate SG-S belongs to the USA300 clonal lineage which has an exceptionally high potential to cause disease, due mainly to a high expression and activity of virulence determinants such as the quorum sensing regulator *agr*, phenol-soluble modulins (PMS), alpha-toxin and PVL [42]–[47]. Surprisingly, all these virulence factors with the exception of *pvl* were down-regulated in the resistant strain. Reduced transcription of the global virulence regulators *agr* [48], *rot* [49], *sarH1* [50] as well as *srrAB* (also involved in the regulation of energy metabolism) [51], and the genetic determinants *hld* and *spa* was also observed in strain SG-R. These findings are consistent with results of other studies with different VISA isolates: acquisition of VISA type resistance seems to be accompanied by attenuation of virulence expression [9], [38], [39], [52].

The prolonged course of disease, frequent treatment failure inspite of extensive antibiotic therapy and frequent relapse observed in most cases of infections with VISA type *S. aureus* are not likely to be simply related to the modestly increased MIC values in the resistant isolates recovered from patients. In wondering about how genetic/biochemical changes detected in VISA type isolates may contribute to these unique clinical features of *S. aureus* infections, it is conceivable that the massive down-regulation of important staphylococcal virulence genes – rather than the modestly increased MIC value – may be the key contributing factor.

Such a decrease in virulence appears to be a direct consequence of the mode of action of vancomycin. In this scenario, vancomycin “traps” the peptidoglycan precursor Lipid II which triggers one of the two-component sensory regulatory systems “assigned” to monitor the status of cell wall biosynthesis in the bacteria. Turning on one or the other of these regulatory systems (such as VraSR, WalKR or GraSR) initiates a cascade of transcriptional events that involve not only cell wall related determinants but also transcription of major virulence genes. Consistent with this model, a reversible down-regulation of virulence genes such as *spa, rot, sarH1* was already described when the vancomycin susceptible strain JH1 was briefly exposed to vancomycin at the MIC value of the isogenic VISA isolate JH9 [39]. In VISA strains such initially reversible down regulation in virulence will become permanent by the appearance of mutations in a relevant genetic determinant such as *yvqF* in the case of the VISA isolate SG-R. Decreased virulence of VISA strains is consistent with observations made in clinical cases of such infections [53]–[56] and was also documented in the rat model [57] and *Galleria mellomella* models [52] of staphylococcal disease.

Our data suggest that VISA strains may employ a “stealth” strategy for invasion of the host. Down-regulation of major virulence determinants such as *agr, rot, sasH1, spa* and others together with the inactivation of an important virulence gene *lspA* detected in the SG-R isolate may allow invading bacteria that seed deep tissues to escape detection by the innate immune system and to persist in body sites such as a damaged heart valve, prosthetic devices or bone tissue where immune surveillance is poor or otherwise compromised.

## Materials and Methods

### Ethics statement

The strains used in this study originated from the University of California San Francisco. The following is their policy on IRB approval. Research on Specimens and Data That Does Not Require CHR Review. The Committee on Human Research (CHR) at UCSF is charged with reviewing all research that involves human subjects http://www.research.ucsf.edu/chr/about/chrProgDesc.asp. However, some research involving only unidentifiable or coded private information or specimens does not qualify as human subjects research and does not require CHR review.

Since the strains were de-identified and analyzed anonymously and the strains, not a human, were studied, this is exempt from human research committee approval.

Preliminary characterization of these isolates was done as described in reference number 11 of this manuscript.

### Strains

A vancomycin susceptible *S. aureus* isolate (vancomycin MIC: 1.5 µg/ml) belonging to the USA300 genetic background was recovered in November 2005 from the blood culture of a 59-year-old male patient admitted to San Francisco General Hospital. This isolate will be referred to as SG-S (for San Francisco General Hospital-Susceptible) throughout the paper. A second isolate of the same clonal type but expressing intermediate resistance to vancomycin (MIC: 3 µg/ml) – named SG-R (for San Francisco General Hospital-Resistant) was recovered from a bone biopsy specimen of the same patient after a 6 week course of vancomycin therapy. Detailed clinical history of the patient and preliminary characterization of the isolates were described recently [11]. A laboratory derived revertant named SG-rev (vancomycin MIC: 1 µg/ml) was obtained by serial passage of SG-R in tryptic soy broth for 15 days in the absence of vancomycin.

USA300 isolates carrying the multicopied carrier plasmid pGC2 [58] were labelled SG-S(pGC2), SG-R(pGC2) and SG-rev(pGC2). Isolates carrying wild type form of *yvqF* or the mutated form of this gene were labeled SG-R(pGC2::*yvqF*wt) or SG-S(pGC2::*yvqF*mut) respectively. Mutants carrying the multicopy plasmid pGC2 containing the wild type *vraSR* genes linked to the *vraSR* operon main promoter region were referred to as SG-rev(pGC2::*vraSR*wt).

### Growth conditions

*S. aureus* strains were grown in tryptic soy broth (TSB) (Difco Laboratories) with aeration at 37°C, on tryptic soy agar plates (TSA) (Difco Laboratories) or on brain heart infusion agar (Difco Laboratories) at 37°C. *Escherichia coli* strains were grown in Luria-Bertani broth (Difco Laboratories) with aeration at 37°C. Chloramphenicol, (20 µg/ml) and ampicillin (100 µg/ml) were used as recommended by the manufacturer (Sigma) for the selection and maintenance of *S. aureus* and *E. coli* transformants, respectively.

### DNA methods

DNA manipulations were performed by standard methods [59]. Restriction enzymes were used as recommended by the manufacturer (Invitrogen). Routine PCR amplification was performed with AmpliTaq DNA Polymerase (Applied Biosystems) Wizard *Plus* Minipreps and Midipreps purification systems (Promega) were used for plasmid extraction. Ligation reactions were performed using T4 Ligase (New England Biolabs). PCR products were cleaned with a QIAquick PCR purification kit and sequenced. Sequencing was performed at Genewiz, Inc, NJ.

### Growth curves

Overnight cultures were diluted in 50 ml TSB to an OD_620_ of 0.02. Cultures were incubated with agitation at 37°C for 5 hours. Increase in OD_620_ was measured every 30 minutes using a Novaspec II Spectrophotometer (Pharmacia, LKB).

### Triton X-100 stimulated autolysis

Cells were grown to mid-exponential phase, chilled in an ice-ethanol bath, harvested, and washed with ice-cold water. Cells were then suspended to an OD_620_ of 1.0 in lysis buffer (50 mM glycine buffer, pH 8.0, containing 0.01% Triton X-100) as previously described [60]. The rate of autolysis was measured at 37°C for 3 h every 15 minutes as a decrease of OD_620_.

### Determination of antibiotic susceptibility

Antibiotic susceptibility level (MIC) to vancomycin, oxacillin, daptomycin and fosfomycin were determined by the E-test following recommendations by the manufacturer (Ab Biodisk). A more detailed evaluation of vancomycin and oxacillin susceptibility was done by population analysis. Linezolid and tigecycline susceptibility were determined by the disk diffusion method following recommendations by the manufacturer (Ab Biodisk).

Susceptibility to moenomycin was determined by tube dilution as previously described [61].

### Population analysis profiles

Population analysis profiles (PAPs) were performed as described previously [62]. Overnight cultures were plated at various dilutions (10^−1^, 10^−2^, 10^−3^ and 10^−5^/10^0^, 10^−1^, 10^−3^ and 10^−5^) on TSA plates containing a series of concentrations of vancomycin (Sigma) or oxacillin (Sigma) and bacterial colonies were counted after incubation of the plates at 37°C for 48 h.

### Analysis of peptidoglycan composition

Cell walls were isolated from exponential phase cells, peptidoglycan purified and hydrolyzed with the M1 muramidase and the resulting muropeptides reduced with borohydride and separated by reverse-phase high-performance liquid chromatography (RP-HPLC) – as previously described [13].

### Transmission Electron Microscopy

One ml aliquots of bacterial cultures were harvested by low-speed centrifugation and fixed with 1 ml of 2.5% (v/v) glutaraldehyde. Electron microscopy was done at the Electron Microscopy Service of The Rockefeller University. Electron micrographs were selected after scanning numerous fields at low power magnification to make sure that photos presented at high power magnification represent “typical” structures.

### Genomic sequence analysis

The genome sequencing was done at the Rockefeller University's Genomics Resource Center using Illumina's Genome Analyzer IIx. Between 3 to 14 million 35 bp reads were generated per isolate. Software, called IdentPoly, was developed in the lab to process the reads, carry out a reference-based assembly, and detect polymorphisms. The reads were trimmed and filtered based on quality scores and then assembled onto the closely related finished genomic sequence of the MRSA strain USA300_TCH1516 [63]–[64]. For each isolate, a mean read coverage of 30–150× was achieved on the reference genome. While a small percentage, <1%, of the reads from each isolate do not map to the reference, these reads are the same amongst the isolates, so it is very likely that these reads are from genomic islands present in the isolates but not the reference.

Polymorphisms were detected using a variant of a Bayesian probabilistic model that was previously described [7]. In contrast to contemporary methods, this model allowed for the detection of polymorphisms >5 bp. The model was rigorously tested on both synthetic and real data harboring known substitutions, insertions, deletions, inversions, etc. ranging from 1–10,000 bp in size.

### Confirmation of genomic mutations

DNA fragments containing the mutations identified by whole genome sequencing were amplified by PCR using the following conditions: 94°C for 4 min; 30 cycles of 94°C for 30 s, 50°C for 30 s, and 72°C for 40 s; and one final extension step of 72°C for 5 min. The primers used for each PCR reaction are described in Table S2. The purified PCR products were cleaned with a QIAquick PCR purification kit and sequenced. Sequencing was performed at Genewiz, Inc, NJ.

### qRT-PCR

The levels of different mRNA transcripts were quantified in the different strains by qRT-PCR. Total RNA was cleaned from DNA contamination using the RNeasy Plus micro kit, which includes a non enzymatic DNA removal step. RT- was performed by using the AffinityScript (Stratagene) in a 20 µl reaction mixture under the following conditions: 10 min at 65°C, 2 h at 42°C and 15 min 70°C by using random hexamers as primers. To quantify cDNA generated by reverse transcription from target RNA, real time PCR with SYBR Green I was performed by using SYBR Green PCR master mix in an ABI Prism 7900 Sequence Detection System (Applied Biosystems). The 10 µl reaction mixture contained 1× iTaq SYBR Green PCR supermix with ROX (BioRad laboratories), forward and reverse primers (each at a concentration of 100 nM), and 5 µl of template (reverse transcription product).The primers were designed by Oligo7 software (Applied Biosystems) and are listed in Table S3. The thermal conditions were: 2 min at 95°C and followed by 40 cycles at 95°C 15 sec, 55°C for 15 sec and 72°C 30 sec. Every sample was run in triplicates. A threshold value for the fluorescence of all samples was set manually. The reaction cycle at which the PCR product exceeds this fluorescence threshold was identified as the threshold cycle (C*_T_*). The C*_T_* was then converted to relative quantity of mRNA by using a standard curve. The standard curve was generated via the qRT-PCR program conditions using serial 2 fold dilutions of genomic DNA. Relative gene expression was expressed as a ratio of specific cDNAs concentration to housekeeping gene (*pta*) concentration.

### Microarrays

Ten micrograms of each RNA sample were reverse transcribed, fragmented, and 3′ biotinylated, following the manufacturer's recommendations for processing antisense prokaryotic arrays (Affymetrix). A total of 1.5 µg of labeled material was then hybridized to an *S. aureus* GeneChip, washed, stained, and scanned as previously described [48], [65]. In total, RNA from at least two independent biological replicates was processed for each strain.

Data obtained from the scans of the microarrays were imported into the Genespring software version GX 11.0 (Agilent). Data were summarized with the GCRMA algorithm [66] and normalized to the quantile in order to reduce chip to chip variation. Baseline transformation was then performed to the mean of all chips. Gene lists were derived with volcano plots, which are constructed using fold change values and p-values. P-values were calculated by the unpaired t–Test with no post hoc correction. Genes that showed a fold change cut off ≥2 accompanied with a p-value cut off ≤0.05 in at least one of the comparisons were selected for further investigation.

All experiments – including growth and autolysis curves, population analysis profiles were repeated at least 2 times in order to assure reproducibility.

### Construction of pGC2::*yvqF*mut, pGC2::*yvqF*wt, and pGC2::*vraSR*wt plasmids

A DNA fragment containing the main promoter region of the *vraSR* operon (upstream from *orf1* [22]) was amplified by PCR using the Phusion High-Fidelity DNA polymerase (New England Biolabs) and primers P_XbaI (5′-AACC**TCTAGA**TGCTCAAATTGAGCATACGG-3′) and PvraSLower (5′-AACTATCACCTTTTATAATAAG-3′). Primer P_XbaI was engineered to carry an XbaI restriction site (underlined in the primer sequence). The following PCR conditions were used: 98°C for 2 min; 25 cycles of 98°C for 10 s, 43°C for 30 s, and 72°C for 20 s; and one final extension step of 72°C for 7 min. The purified PCR product was cleaned, digested with XbaI (Invitrogen), and phosphorylated with a T4 Polynucleotide Kinase (New England Biolabs).

The wild type forms of *yvqF*/*vraSR* genes and the mutated form of *yvqF* gene were amplified from the SG-S and SG-R chromosomal DNAs, respectively, using the Phusion High-Fidelity DNA polymerase (New England Biolabs) and primers

yvqF300UP (5′-ATGACACACAAATATATATCAACGCAAATG-3′)/yvqF300Lower_HindIII (5′-AACC**AAGCTT**TCATCGATAAATCACCTCTA-3′) and VraSUP (5′-ATGAACCACTACATTAGAA CAATTGGTTCA-3′)/VraRLower_HindIII (**5′** – CCCC**AAGCTT**CTATTGAATTAAATTATGTT-**3′** ), respectively. Primers yvqF300Lower_HindIII and VraRLower_HindIII were engineered to carry a HindIII restriction site (underlined in the primer sequences). The following PCR conditions were used: 98°C for 2 min; 25 cycles of 98°C for 10 s, 43°C for 30 s, and 72°C for 30 s (amplification of the *yvqF* gene) and 98°C for 2 min; 25 cycles of 98°C for 10 s, 43°C for 30 s, and 72°C for 1 min (amplification of the *vraSR* genes); and one final extension step of 72°C for 7 min. The purified PCR products were cleaned, digested with HindIII (Invitrogen), fused separately with the *vraSR* phosphorylated promoter region and linked to the pGC2 digested with XbaI and HindIII. The ligation mixture was used to transform *E. coli* DH5α (Invitrogen) competent cells to obtain plasmids pGC2::*yvqF*mut, pGC2::*yvqF*wt, and pGC2::*vraSR*wt. The correct sequences of the three constructs were confirmed.

### Construction of SG-S, SG-R and SG-rev mutants complemented with *yvqF*mut, *yvqF*wt, and *vraSR*wt

Plasmids pGC2::*yvqF*mut, pGC2::*yvqF*wt, and pGC2::*vraSR*wt were introduced separately into *S. aureus* RN4220 electrocompetent cells by electroporation with a Gene pulser apparatus (Biorad) essentially as previously described [67]. The transformation mixture was plated on TSA containing chloramphenicol (20 µg/ml). Plasmids pGC2::*yvqF*mut, pGC2::*yvqF*wt, and pGC2::*vraSR*wt were extracted and then electroporated separately into strains SG-S, SG-R and SG-rev, respectively, as described previously [68]. The transformation mixture was plated on BHI containing chloramphenicol (20 µg/ml) and two colonies resistant to chloramphenicol from each different background were selected for further assays.

## Supporting Information

Figure S1 Heat map of differentially expressed genes in SG-R compared to SG-S, SG-R compared to SG-rev, and SG-rev compared to SG-S. *COL genome annotation.
(PDF)

Figure S2 **Transcription of genes in strains SG-S(pGC2), SG-R(pGC2) and SG-R(pGC2::yvqF wt).** **A. Transcription of genes of the VraSR regulon.** The expression of each mRNA was determined by qRT-PCR. As internal control, the expression was also determined in each sample for the pta mRNA. Different assays provided evidence that *lytM* is most probably an additional a member of the VraSR regulon (data not shown). orf 2221 encodes for an hypothetical protein. **B. Transcription of genes with a variety of metabolic functions.** Transcription of *lctE* (energy metabolism), *acpD* (fatty acid and phospholipid metabolism), *sodM* (detoxification), *sspB* (protein fate), *sirA* (inorganic ion transport and metabolism), *narK* (energy metabolism), and *sptC* (transport and binding proteins) was compared in strains SG-S(pGC2), SG-R(pGC2) and SG-R(pGC2::*yvqF* wt). The expression of each mRNA was determined by qRT-PCR. As internal control, the expression was also determined in each sample for the *pta* mRNA.
(PDF)

Table S1 **Genes with significant expression changes in SG-R compared to SG-S, and SG-R compared to SG-rev and SG-rev compared with SG-S.** The p-values that are not listed are ≤0.05. Genes listed in bold were found up-regulated or down-regulated in the VISA strain JH9 when compared with the parental susceptible strain JH1 [1]; Genes identified with a star are considered members of the VraSR regulon according to Kuroda *et al.* [2]. (a) Genes with altered transcription when *S. aureus* is treated with inhibitory concentrations of oxacillin, D-cycloserine and bacitracin [3]. (b) members of the cell wall stimulon of *S.aureus* [3]. SA – N315 genome ORF annotation; SACOL – COL genome ORF annotation; SAUSA300 – USA300 genome ORF annotation; SAV – MU50 genome ORF annotation. [**1**] McAleese F, Wu SW, Sieradzki K, Dunman P, Murphy E, et al. (2006) Overexpression of genes of the cell wall stimulon in clinical isolates of *Staphylococcus aureus* exhibiting vancomycin-intermediate- *S. aureus*-type resistance to vancomycin. J Bacteriol 188: 1120–1133. [**2**] Kuroda M, Kuroda H, Oshima T, Takeuchi F, Mori H, et al. (2003) Two-component system VraSR positively modulates the regulation of cell-wall biosynthesis pathway in *Staphylococcus aureus*. Mol Microbiol 49: 807-821. [**3**] Utaida S, Dunman PM, Macapagal D, Murphy E, Projan SJ, et al. (2003) Genome-wide transcriptional profiling of the response of *Staphylococcus aureus* to cell-wall-active antibiotics reveals a cell-wall-stress stimulon. Microbiology 149: 2719–2732.
(DOC)

Table S2 **Primers used in sequencing.**
(DOC)

Table S3 **Primers used in the qRT-PCR studies.**
(DOC)
