# Supplementary material for: Genetic Pathway in Acquisition and Loss of Vancomycin Resistance in a Methicillin Resistant Staphylococcus aureus (MRSA) Strain of Clonal Type USA300
Source: PLoS Pathog. 2012 Feb 2;8(2):e1002505. doi: 10.1371/journal.ppat.1002505 (PMC3271070; doi:10.1371/journal.ppat.1002505)
Supplement: Figure S1 — Heat map of differentially expressed genes in SG-R compared to SG-S, SG-R compared to SG-rev, and SG-rev compared to SG-S. *COL genome annotation. (PDF) [file ppat.1002505.s001.pdf]

ORF annotation	SG-R / SG-S	SG-R / SG-rev	SG-rev / SG-S
SAS052			
SAS079			
SA0352			
SA0354			
SA0497			
SA0498			
SA0502			
SA0503			
SA1112			
SA1414			
SA1473			
SA1502			
SA1503			
SA1504			
SA1083			
SA2016			
SA2033			
SA2035			
SA2036			
SA2037			
SA0266			
SA0293			
SA0294			
SA0295			
SA0531			
SA0616			
SA0617			
SA0640			
SA1183			
SA1257			
SA1815			
SA1987			
SA2205			
SA2156			
SA1270			
SA2239			
SA2291			
SA2302			
SA2486			
SA2489			
SA0825			
SA0826			
SA1253			
SA1549			
SA1725			
SA1758			
SA1256			
SA1659			
SA0010			
SA0180			
SA2135			
SA1163			
SA0411			
SA0911			
SA0937			
SA0938			
SA0996			
SA1517			
SA1801			
SA2146			
SA2176			
SA2185			
SA2220			
SA0232			
SA1255			
SA2378			
SA2395			
SAS065			
SA0107			
SA0270			
SA0745			
SA1007			
SA1322			
SA1323			
SA1583			
SA1752			
SA1755			
SA1812			
SA1813			
SAS020			

ORF annotation	SG-R / SG-S	SG-R / SG-rev	SG-rev / SG-S
SA2097			
SA2206			
SA1186*			
SA1187*			
SA0039			
SA0108			
SA0836			
SA0904			
SA0949			
SA1195			
SA2092			
SA1665			
SA1804			
SA2103			
SA2108			
SA2296			
SA0038			
SA0127			
SA0205			
SA0243			
SA0265			
SA0909			
SA1474			
SA1691			
SA1926			
SA1043*			
SA0129			
SA0341			
SA0591			
SA2006			
SA2303			
SA0895			
SA0964			
SA0965			
SA1491			
SA1494			
SA1495			
SA2186			
SA0110			
SA0111			
SA0566			
SA0927			
SA0928			
SA1016			
SA0241			
SA2490			
SA0562			
SA1365			
SA2183			
SA2187			
SA2189			
SA2312			
SA2413			
SA2024*			
SA1701			
SA1700			
SA2023*			
SA2025*			
SA2026*			
SA2326			
SA0189			
SA0353			
SA0746			
SA1282			
SA1929			
SA0022			
SA0182			
SA0325			
SA2053			
SA1269			
SA0128			
SAV1991			
SA0091			
SA0204			
SA0309			
SA1073			
SA1074			
SA2480			
SA0007			
SAS016			

ORF annotation	SG-R / SG-S	SG-R / SG-rev	SG-rev / SG-S
SAS028			
SAS037			
SA0175			
SA0181			
SA0183			
SA0213			
SA0228			
SA0231			
SA0267			
SA0269			
SA0271			
SA0395			
SA0423			
SA0530			
SA0543			
SA0551			
SA0552			
SAS0630			
SA0645			
SA0666			
SA0667			
SA0681			
SA0755			
SA0817			
SA0824			
SA0882			
SA0914			
SA0959			
SA0966			
SA1002			
SA1015			
SA1017			
SA1020			
SA1040			
SA1049			
SA1152			
SA1254			
SA1265			
SA1316			
SA1472			
SA1476			
SA1514			
SA1576			
SA1617			
SA1702			
SA1703			
SA1705			
SA1712			
SA1738			
SA1803			
SA1840			
SA1898			
SA1942			
SA2001			
SA2062			
SA2113			
SA2191			
SA2192			
SA2221			
SA2268			
SA2297			
SA2310			
SA2328			
SA2329			
SA2343			
SA2377			
SA2431			
SA2432			
SA2479			
SA2481			
SA2487			
SA0850*			
SA1042*			
SA1345*			
SA1346*			
SA1347*			
SA1999*			
SA2433*			
SA2559*			

-4	-3	-2	-1	-0.5	0	0.5	1	1.5	2	3	4
