# Supplementary figures and images for: Genetic Pathway in Acquisition and Loss of Vancomycin Resistance in a Methicillin Resistant Staphylococcus aureus (MRSA) Strain of Clonal Type USA300
Source: PLoS Pathog. 2012 Feb 2;8(2):e1002505. doi: 10.1371/journal.ppat.1002505 (PMC3271070; doi:10.1371/journal.ppat.1002505)

**A****Gene members of the *VraSR* regulon**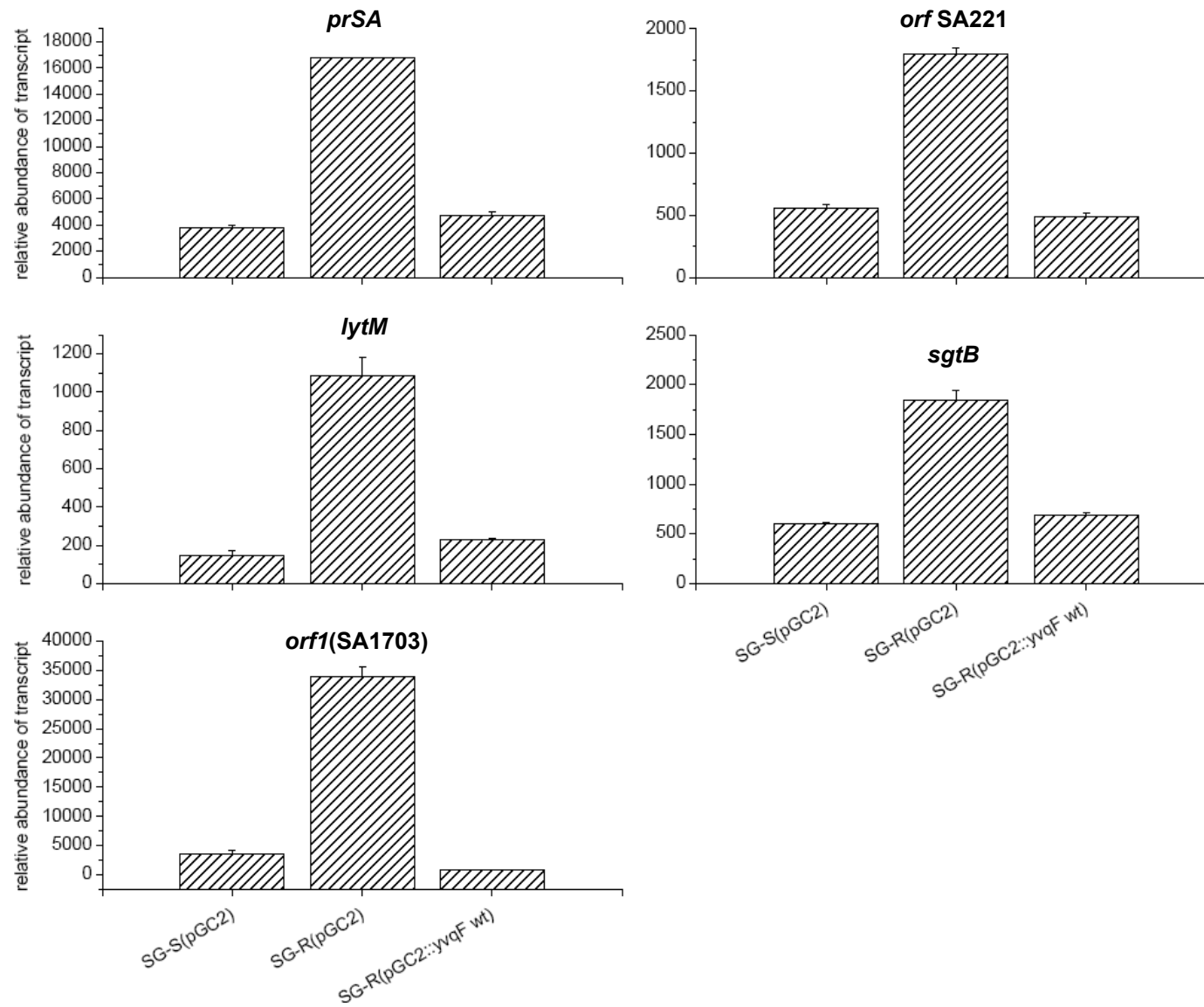

**B****Genes with a variety of metabolic functions**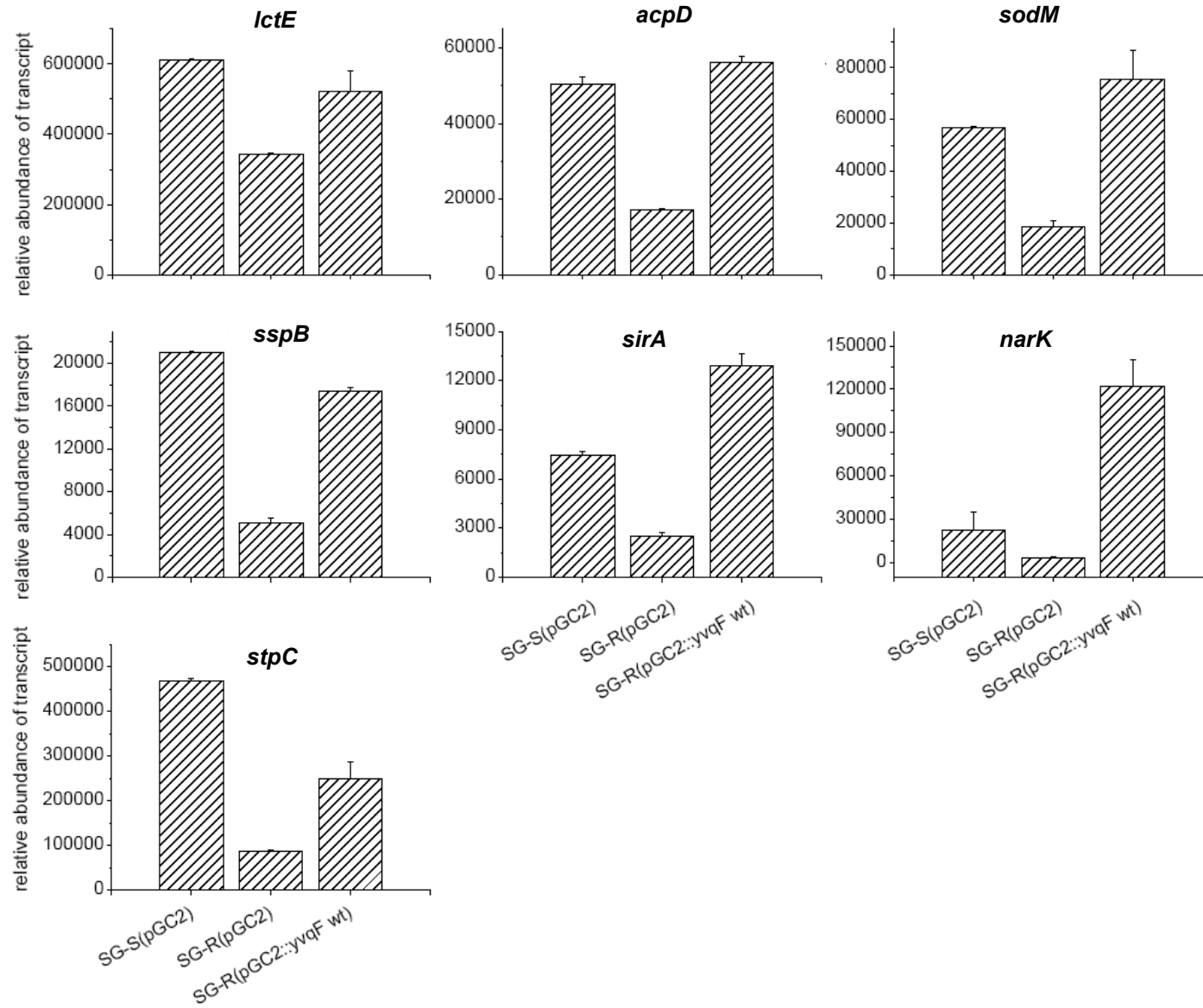

Supplement: Figure S2 — Transcription of genes in strains SG-S(pGC2), SG-R(pGC2) and SG-R(pGC2::yvqF wt). A. Transcription of genes of the VraSR regulon. The expression of each mRNA was determined by qRT-PCR. As internal control, the expression was also determined in each sample for the pta mRNA. Different assays provided evidence that lytM is most probably an additional a member of the VraSR regulon (data not shown). orf 2221 encodes for an hypothetical protein. B. Transcription of genes with a variety of metabolic functions. Transcription of lctE (energy metabolism), acpD (fatty acid and phospholipid metabolism), sodM (detoxification), sspB (protein fate), sirA (inorganic ion transport and metabolism), narK (energy metabolism), and sptC (transport and binding proteins) was compared in strains SG-S(pGC2), SG-R(pGC2) and SG-R(pGC2::yvqF wt). The expression of each mRNA was determined by qRT-PCR. As internal control, the expression was also determined in each sample for the pta mRNA. (PDF) [file ppat.1002505.s002.pdf]
