# Supplementary material for: Genetic Pathway in Acquisition and Loss of Vancomycin Resistance in a Methicillin Resistant Staphylococcus aureus (MRSA) Strain of Clonal Type USA300
Source: PLoS Pathog. 2012 Feb 2;8(2):e1002505. doi: 10.1371/journal.ppat.1002505 (PMC3271070; doi:10.1371/journal.ppat.1002505)
Supplement: Table S1 — Genes with significant expression changes in SG-R compared to SG-S, and SG-R compared to SG-rev and SG-rev compared with SG-S. The p-values that are not listed are ≤0.05. Genes listed in bold were found up-regulated or down-regulated in the VISA strain JH9 when compared with the parental susceptible strain JH1 [1]; Genes identified with a star are considered members of the VraSR regulon according to Kuroda et al. [2]. (a) Genes with altered transcription when S. aureus is treated with inhibitory concentrations of oxacillin, D-cycloserine and bacitracin [3]. (b) members of the cell wall stimulon of S.aureus [3]. SA – N315 genome ORF annotation; SACOL – COL genome ORF annotation; SAUSA300 – USA300 genome ORF annotation; SAV – MU50 genome ORF annotation. [1] McAleese F, Wu SW, Sieradzki K, Dunman P, Murphy E, et al. (2006) Overexpression of genes of the cell wall stimulon in clinical isolates of Staphylococcus aureus exhibiting vancomycin-intermediate- S. aureus-type resistance to vancomycin. J Bacteriol 188: 1120–1133. [2] Kuroda M, Kuroda H, Oshima T, Takeuchi F, Mori H, et al. (2003) Two-component system VraSR positively modulates the regulation of cell-wall biosynthesis pathway in Staphylococcus aureus. Mol Microbiol 49: 807-821. [3] Utaida S, Dunman PM, Macapagal D, Murphy E, Projan SJ, et al. (2003) Genome-wide transcriptional profiling of the response of Staphylococcus aureus to cell-wall-active antibiotics reveals a cell-wall-stress stimulon. Microbiology 149: 2719–2732. (DOC) [file ppat.1002505.s003.doc]

|  |  | |  | **Fold change** | | |  |
| --- | --- | --- | --- | --- | --- | --- | --- |
| **ORF annotation** | **gene** | | **Product of putative function** | **SG-R / SG-S** | **SG-R / SG-rev** | **SG-rev / SG-S** | **Functional category** |
| SAS052 | *rpsD* | | 30S ribosomal protein S4 | - 2.24 | - 2.00 |  | Ribosomal proteins: Synthesis and modification |
| SAS079 | *rpsN* | | 30S ribosomal protein S14 | - 3.42 | - 3.28 |  | Ribosomal proteins: Synthesis and modification |
| SA0352 | *rpSG* | | 30S ribosomal protein S6 | - 2.26 | - 1.98 |  | Ribosomal proteins: Synthesis and modification |
| SA0354 | *rpsR* | | 30S ribosomal protein S18 | - 2.11 | - 1.83 |  | Ribosomal proteins: Synthesis and modification |
| SA0497 | *rplJ* | | 50S ribosomal protein L10 | - 2.13 | - 2.20 |  | Ribosomal proteins: Synthesis and modification |
| SA0498 | *rplL* | | 50S ribosomal protein L7/L12 | - 2.16 | - 2.18 |  | Ribosomal proteins: Synthesis and modification |
| SA0502 |  | | Hypothetical protein, similar to ribosomal protein L7Ae-like | - 2.05 | (- 1.86; p = 0.123) |  | Ribosomal proteins: Synthesis and modification |
| SA0503 | *rpsL* | | 30S ribosomal protein S12 | - 2.30 | (- 1.61; p = 0.336) |  | Ribosomal proteins: Synthesis and modification |
| SA1112 | *infB* | | Translation initiation factor IF-2 | - 2.12 | - 1.59 |  | Protein synthesis: Translation factors |
| SA1414 | *rpsT* | | 30S ribosomal protein S20 | - 1.74 | - 2.02 |  | Ribosomal proteins: Synthesis and modification |
| SA1473 | *rplU* | | 50S ribosomal protein L21 | - 2.19 | - 2.08 |  | Ribosomal proteins: Synthesis and modification |
| SA1502 | *rplT* | | 50S ribosomal protein L20 | - 2.89 | - 2..35 |  | Ribosomal proteins: Synthesis and modification |
| SA1503 | *rpmI* | | 50S ribosomal protein L35 | - 2.45 | - 2.09 |  | Ribosomal proteins: Synthesis and modification |
| SA1504^a^ | *infC* | | Translation initiation factor IF-3 InfC | - 2.62 | - 1.93 |  | Protein synthesis: Translation factors |
| SA1083 | *trmD* | | tRNA-(guanine-N1)-mehtyltranSGerase | - 2.25 | (- 1.58; p = 0.127) |  | Protein synthesis: tRNA and rRNA base modification |
| SA2016 | *rpsI* | | 30S ribosomal protein S9 | - 2.13 | - 1.76 |  | Ribosomal proteins: Synthesis and modification |
| SA2033 | *rplF* | | 50S ribosomal protein L6 | - 2.46 | - 2.29 |  | Ribosomal proteins: Synthesis and modification |
| SA2035 | *rplE* | | 50S ribosomal protein L5 | - 2.33 | - 2.30 |  | Ribosomal proteins: Synthesis and modification |
|  |  | |  | **Fold change** | | |  |
| **ORF annotation** | **Gene** | | **Product of putative function** | **SG-R / SG-S** | **SG-R / SG-rev** | **SG-rev / SG-S** | **Functional category** |
| SA2036 | *rplX* | | 50S ribosomal protein L24 | - 2.44 | - 2.45 |  | Ribosomal proteins: Synthesis and modification |
| SA2037 | *rplN* | | 50S ribosomal protein L14 | - 2.16 | - 2.70 |  | Ribosomal proteins: Synthesis and modification |
| SA0266 |  | | Conserved hypothetical protein | - 2.02 | - 1.86 |  | Transport and binding proteins |
| SA0293 |  | | Hypothetical protein, similar to formate transporter NirC | - 11.52 | - 9.32 |  | Transport and binding proteins: Anions |
| SA0294 |  | | Hypothetical protein, similar to branched-chain amino acid uptake carrier | - 2.80 | - 2.79 |  | Transport and binding proteins: Amino acids, peptides and amines |
| **SA0295** |  | | Hypothetical protein, similar to outer membrane protein precursor | - 4.67 | - 4.63 |  | Transport and binding proteins |
| SA0531* | *proP* | | Proline/betaine transporter homologue | (1.85; p = 0.058) | 2.22 |  | Transport and binding proteins |
| **SA0616** | *vraF* | | ABC transporter ATP-binding protein | 2.11 | - 1.23 | 2.59 | Transport and binding proteins |
| **SA0617** | *vraG* | | ABC transporter permease | 2.59 | - 1.23 | 3.18 | Transport and binding proteins |
| SA0640 |  | | Hypothetical protein, similar to ABC transporter required for expression of cytochrome bd | - 2.03 | - 1.85 |  | Transport and binding proteins |
| SA1183***** | *opuD* | | Glycine betaine transporter | - 2.72 | - 2.96 |  | Transport and binding proteins: Amino acids, peptides and amines |
| SA1257^a,b^ | *mrsA* | | Peptide methionine sulfoxide reductase | 3.45 | 5.61 |  | Transport and binding proteins: Carbohydrates, organic alcohols, and acids |
| SA1815 |  | | Hypothetical protein, similar to Na+-transporting ATP synthase | - 2.17 | - 2.03 |  | Transport and binding proteins: Cations and iron carrying compounds |
| SA1987 |  | | Glycine betaine transporter OpuD homolog | 1.54 | 2.21 |  | Transport and binding proteins: Amino acids, peptides and amines |
| SA2205 |  | | Conserved hypothetical protein | -2.02 | - 1.70 |  | Transport and binding proteins: Cations and iron carrying compounds |
| **SA2156**^a^ |  | | L-lactate permease lctP homolog | - 12.73 | - 11.48 |  | Transport and binding proteins: Carbohydrates, organic alcohols, and acids |
| SA1270 |  | | Hypothetical protein, similar to amino acid pearmease | - 3.45 | (- 2.95; p = 0.175) |  | Transport and binding proteins: Amino acids, peptides and amines |
| SA2239 |  | | Hypothetical protein, similar to Amino acid transporter | 2.75 | 3.03 |  | Transport and binding proteins: Amino acids, peptides and amines |
| SA**2291** |  | | Fibronectin-binding protein homolog | 2.53 | 2.18 |  | Transport and binding proteins |
| **SA2302** | *stpC* | | ABC transporter | - 57.87 | - 40.7 |  | Transport and binding proteins |
| SA2486 |  | | 2-oxoglutarate/malate translocator homolog | - 2.62 | - 2.19 |  | Transport and binding proteins: Anions |
| SA2489^a,b^ |  | | Hypothetical protein, similar to high-affinity nickel-transport protein | 5.34 | 4.97 |  | Transport and binding proteins: Cations and iron carrying compounds |
|  |  | |  | **Fold change** | | |  |
| **ORF annotation** | **Gene** | | **Product of putative function** | **SG-R / SG-S** | **SG-R / SG-rev** | **SG-rev / SG-S** | **Functional category** |
| SA0825* | *spsA* | | Type-I signal peptidase | 2.85 | 3.48 |  | Protein fate: Protein and peptide secretion and trafficking |
| SA0826^a,b^ | *spsB* | | Type 1 signal peptidase 1B | 2.28 | 2.47 |  | Protein fate: Protein and peptide secretion and trafficking |
| SA1253* | *ctpA* | | Carboxy-terminal processing proteinase CtpA | 1.50 | 2.02 |  | Protein fate: Degradation of proteins, peptides, and glycopeptides |
| SA1549*^a,b^ | *htrA* | | Heat shock protein homolog, similar to serine proteinase | 2.63 | 4.51 |  | Protein fate: Degradation of proteins, peptides, and glycopeptides |
| **SA1725** | *sspB* | | Staphopain cysteine protease | - 7.05 | - 4.10 |  | Protein fate: Degradation of proteins, peptides, and glycopeptides / Pathogenesis |
| SA1758 | *sak* | | Staphylokinase precursor | - 3.83 | - 4.95 |  | Protein fate: Degradation of proteins, peptides, and glycopeptides |
| SA1256 ^a,b^ |  | Methionine sulfoxide reductase B (MsrB) | | 3.23 | 4.45 |  | Post-translational modification, protein turnover and chaperones |
| **SA1659***^a,b^ | *prsA* | | Peptidyl-prolyl cis/trans isomerase homolog | 3.09 | 4.22 |  | Post-translational modification, protein turnover and chaperones |
| **SA0010** | *azlC* | | Putative amino acid permease | - 2.12 | - 2.29 |  | Amino acid transport and metabolism |
| **SA0180** | *brnQ* | | Branched-chain amino acid permease | - 2.14 | (- 2.11; p= 0.081) |  | Amino acid transport and metabolism |
| **SA2135** | *gltS* | | Sodium/glutamate symporter | - 4.20 | - 5.42 |  | Amino acid transport and metabolism |
| SA1163 |  | | Aspartate kinase homolog | -2.48 | - 1.75 |  | Amino acid biosynthesis: Aspartate family |
| SA0411 | *ndhF* | | NADH dehydrogenase subunit 5 | - 2.47 | (- 2.32; p= 0.071) |  | Energy metabolism: Electron transport |
| SA0911^a^ | *qoxC* | | Quinol oxidase polypeptide III QoxC | - 2.15 | - 1.90 |  | Energy metabolism: Electron transport |
| SA0937 |  | | Cytochrome *d* ubiquinol oxidase subunit 1 homolog | - 2.53 | (- 2.28; p = 0.22) |  | Energy metabolism: Electron transport |
| SA0938 |  | | Sytochrome D ubiquinol oxidase subunit II homolog | - 2.62 | (- 2.65; p= 0.177) |  | Energy metabolism: Electron transport |
| SA0996 | *sdhB* | | Succinate dehydrogenase iron-sulfur protein subunit | - 2.20 | - 1.76 |  | Energy metabolism: TCA cycle |
| SA1517 | *citC* | | Isocitrate dehyrogenase | 2.15 | 1.96 |  | Energy metabolism: TCA cycle |
| SA1801 |  | | Anti repressor | 2.68 | 1.94 |  | Energy metabolism: Electron transport |
| SA2146*^a,b^ | *tcaA* | | Teicoplanin resistance-associated protein | 4.65 | 5.55 |  | Energy metabolism: Electron transport |
| SA2176^a^ | *narK* | | Nitrite extrusion protein | - 27.82 | - 20.32 |  | Energy metabolism: Electron transport |
| SA2185^a^ | *narG* | | Respiratory nitrate reductase alpha chain | - 3.18 | (- 2.44; p= 0.269) |  | Energy metabolism: Anaerobic |
| SA2220*^a,b^ |  | | Hypothetical protein | (2.60; p = 0.10) | 3.31 |  | Energy metabolism: Glycolysis/gluconeogenesis |

|  |  |  | **Fold change** | | |  |
| --- | --- | --- | --- | --- | --- | --- |
| **ORF annotation** | **Gene** | **Product of putative function** | **SG-R / SG-S** | **SG-R / SG-rev** | **SG-rev / SG-S** | **Functional category** |
| SA0232^a^ | *lctE* | L-lactate dehydrogenase | - 104.54 | - 74.07 |  | Energy metabolism: Glycolysis/gluconeogenesis |
| SA1255*^a,b^ |  | PTS system, glucose-specific enzyme II, A component | 2.94 | 4.00 |  | Energy metabolism: Glycolysis / Gluconeogenesis |
| SA2378 |  | Conserved hypothetical protein | 2.84 | 2.71 |  | Energy metabolism: Pyruvate dehydrogenase |
| SA2395 |  | L-lactate dehydrogenase | - 2.86 | - 2.56 |  | Energy metabolism |
| **SAS065** | *hld* | Delta-hemolysin | - 2.06 | - 2.05 |  | Pathogenesis |
| **SA0107**^a^ | *spa* | Immunoglobulin G binding protein A precursor | - 125.74 | - 100.55 |  | Pathogenesis |
| **SA0270** |  | Hypothetical protein, similar to secretory antigen precursor SsaA | - 2.40 | - 2.52 |  | Pathogenesis |
| **SA0745** |  | Hypothetical protein, similar to extracellular matrix and plasma binding | - 1.87 | - 2.08 |  | Pathogenesis |
| SA1007 |  | Alpha-Hemolysin | - 6.50 | - 4.39 |  | Pathogenesis |
| SA1322 | *srrB* | Staphylococcal respiratory response protein SrrB | - 2.28 | - 1.81 |  | Pathogenesis |
| SA1323 | *srrA* | Staphylococcal respiratory response protein SrrA | - 2.78 | - 2.25 |  | Pathogenesis |
| SA1583 | *rot* | Repressor of toxins Rot | - 2.91 | - 2.94 |  | Pathogenesis |
| SA1752 | Truncated (*hlb*) | Truncated beta-hemolysin | 3.09 | 2.42 |  | Pathogenesis |
| SA1755 |  | Chemotaxis-inhibiting protein CHIPS | - 3.27 | - 4.25 |  | Pathogenesis |
| SA1812 |  | Uncharacterized leukocidin-like protein 1 precursor | - 4.26 | - 3.98 |  | Pathogenesis |
| SA1813 |  | Hypothetical protein, similar to leukocidin chain LukM | - 6.34 | (- 4.66 ; p = 0.052) |  | Pathogenesis |
| **SA2097** |  | Hypothetical protein, similar to secretory antigen precursor SsaA | 4.19 | 3.34 |  | Pathogenesis |
| **SA2206** | *sbi* | IgG-binding protein SBI | - 2.51 | - 2.11 |  | Pathogenesis |
| **SACOL1186 / SAUSA300_1067** |  | Antibacterial protein  (phenol soluble modulin β1) | -11.34 | - 12.80 |  | Pathogenesis |
| **SACOL1187 /**  **SAUSA300_1068** |  | Antibacterial protein  (phenol soluble modulin β2) | - 27.42 | - 27.11 |  | Pathogenesis |
| **SA0039** | *mecR* | Methicillin resistance regulatory protein | - 2.05 | - 1.87 |  | Regulatory functions |
| **SA0108** | *sarH1* | Staphylococcal accessory regulator A homologue | - 5.39 | - 5.38 |  | Regulatory functions: DNA interactions |
| SA0836 |  | Hypothetical protein, similar to transcription regulator LysR | - 3.98 | - 3.47 |  | Regulatory functions: DNA interactions |
| **SA0904** |  | Hypothetical protein, probable ATL autolysin transcription regulator | - 6.15 | - 4.97 |  | Regulatory functions: DNA interactions |
|  |  |  | **Fold change** | | |  |
| **ORF annotation** | **Gene** | **Product of putative function** | **SG-R / SG-S** | **SG-R / SG-rev** | **SG-rev / SG-S** | **Functional category** |
| SA0949 |  | Conserved hypothetical protein | - 2.09 | - 1.83 |  | Regulatory functions: DNA interactions |
| SA1195^a,b^ | *msrR* | Peptide methionine sulfoxide reductase regulator MsrR | 2.92 | 4.11 |  | Regulatory functions |
| **SA2092** |  | Hypothetical protein similar to transcriptional regulator | - 2.82 | - 1.81 | - 1.55 | Regulatory functions: DNA interactions |
| SA1665 |  | Hypothetical protein | - 2.42 | - 2.30 |  | Regulatory functions: DNA interactions |
| SA1804 |  | Putative phage transcriptional regulator | 4.39 | 4.46 |  | Regulatory functions: DNA interactions |
| **SA2103***^a,b^ |  | Hypothetical protein, similar to lyt divergon expression attenuator LytR | 6.75 | 8.74 |  | Regulatory functions: DNA interactions |
| **SA2108** |  | Hypothetical protein, similar to transcription regulator, RpiR | - 2.53 | - 2.64 |  | Regulatory functions: DNA interactions |
| **SA2296*** |  | Hypothetical protein, similar to transcriptional regulator, MerR | 2.82 | 3.12 |  | Regulatory functions: DNA interactions |
| **SA0038** | *mecA* | Penicillin-binding protein 2a | - 3.46 | - 4.13 |  | Cell envelope biogenesis |
| SA0127 |  | Hypothetical protein, similar to capsular polysaccharide synthesis protein 14L | - 2.01 | - 1.61 |  | Cell envelope: Biosynthesis and degradation of surface polysaccharides and lipopolysaccharides |
| **SA0205** |  | Hypothetical protein, similar to lysostaphin precursor | 2.03 | (1.93; p=0.058) |  | Cell envelope biogenesis |
| **SA0243** | *tagB* | Hypothetical protein, similar to teichoic acid biosynthesis protein B | - 3.12 | - 2.32 |  | Cell envelope biogenesis |
| **SA0265** | *lytM* | Peptidoglycan hydrolase | 4.93 | 6.45 |  | Cell envelope biogenesis |
| SA0909*^a,b^ | *fmtA* | Fmt, autolysis and methicillin resistant-related protein | 1.65 | 2.00 |  | Cell envelope: Biosynthesis and degradation of murein sacculus and peptidoglycan |
| SA1474 |  | Hypothetical protein, similar to cell shape determinant *mreD* | - 2.01 | (- 1.40; p= 0.217) |  | Cell envelope: Biosynthesis and degradation of murein sacculus and peptidoglycan |
| **SA1691***^a,b^ | *sgtB* | Monofunctional glycosyltranSGerase | 5.88 | 7.01 |  | Cell envelope biogenesis |
| SA1926*^a,b^ | *murZ* | UDP-*N*-acetylglucosamine 1-carboxylvinyl tranSGerase 2 | 2.53 | 2.56 |  | Cell envelope biogenesis |
| SAUSA300_0939 / SACOL1043 |  | Glycosyl tranSGerase, group 1 family protein (GT1_gtfA_like) | - 5.95 | - 4.82 |  | Cell envelope biogenesis |
| SA0129 |  | Hypothetical protein | - 13.54 | - 15.36 |  | Cell envelope |
| SA0341 |  | Hypothetical protein, similar to low-temperature requirement A protein (LtrA) | - 3.07 | - 2.80 |  | Cell envelope |
| **SA0591**^a,b^ |  | Hypothetical protein | 7.16 | 8.41 |  | Cell envelope |
| **SA2006** |  | Putative MHC class II analog | 5.13 | 4.37 |  | Cell envelope |
|  |  |  | **Fold change** | | |  |
| **ORF annotation** | **Gene** | **Product of putative function** | **SG-R / SG-S** | **SG-R / SG-rev** | **SG-rev / SG-S** | **Functional category** |
| **SA2303**^a^ | *smpC* | Hypothetical protein, similar to membrane spanning protein | - 31.35 | - 27.84 |  | Cell envelope |
| SA0895 |  | Hypothetical protein, similar to menaquinone-specific isochorismate synthase | - 1.78 | - 2.03 |  | Biosynthesis of cofactors, prosthetic groups, and carriers: Menaquinone and ubiquinone |
| SA0964 |  | Hypothetical protein, similar to heme synthase | - 2.92 | - 2.92 |  | Biosynthesis of cofactors, prosthetic groups, and carriers: Heme, porphyrin, and cobalamin |
| SA0965^a^ | *ctaB* | Cytochrome caa3 oxidase  (assembly factor) homolog | - 2.44 | - 2.34 |  | Biosynthesis of cofactors, prosthetic groups, and carriers: Heme, porphyrin, and cobalamin |
| SA1491 | *hemL* | Glutamate-1-semialdehyde 2,1-aminomutase | - 2.02 | (- 1.75; p= 0.091) |  | Biosynthesis of cofactors, prosthetic groups, and carriers: Heme, porphyrin, and cobalamin |
| SA1494 | *hemC* | Porphobilinogen deaminase | - 2.72 | - 2.23 |  | Biosynthesis of cofactors, prosthetic groups, and carriers: Heme, porphyrin, and cobalamin |
| SA1495 | *hemX* | HemA concentration negative effector hemX | - 2.32 | (- 1.85; p= 0.069) |  | Biosynthesis of cofactors, prosthetic groups, and carriers: Heme, porphyrin, and cobalamin |
| SA2186^a^ | *naSG* | uroporphyrin-III C-methyl tranSGerase | - 2.40 | (- 2.036; p= 0.204) |  | Biosynthesis of cofactors, prosthetic groups, and carriers: Heme, porphyrin, and cobalamin |
| **SA0110** | *sirB* | Iron compound ABC transporter | - 2.15 | - 1.56 |  | Inorganic ion transport and metabolism |
| **SA0111** | *sirA* | Iron compound ABC transporter | - 3.77 | - 3.28 |  | Inorganic ion transport and metabolism |
| **SA0566** |  | Hypothetical protein, similar to iron-binding protein | - 2.00 | (- 1.96; p =0.061) |  | Inorganic ion transport and metabolism |
| **SA0927** |  | Hypothetical protein, similar to Cobalt transport protein | - 2.20 | - 1.67 |  | Inorganic ion transport and metabolism |
| **SA0928** |  | Hypothetical protein, similar to cation ABC transporter | - 2.24 | - 1.84 |  | Inorganic ion transport and metabolism |
| **SA1016** |  | Hypothetical protein | - 2.27 | - 2.18 |  | Inorganic ion transport and metabolism |
| **SA0241** | *ispD* | Hypothetical protein similar to 4-Diphosphocytidyl-2-methyl-D-erithritol synthase | - 2.12 | - 1.64 |  | Biosynthesis of secondary metabolites:Terpenoid backbone biosynthesis) |
| SA2490^a,b^ |  | Hypothetical protein, similar to  N-hydroxyarylamine O-acetyltranSGerase | 2.77 | 2.61 |  | Biosynthesis of secondary metabolites |
| SA0562^a^ | *adh1* | Alcohol dehydrogenase I | - 10.15 | (- 5.97; p = 0.133) |  | Central intermediary metabolism |

|  |  |  | **Fold change** | | |  |
| --- | --- | --- | --- | --- | --- | --- |
| **ORF annotation** | **Gene** | **Product of putative function** | **SG-R / SG-S** | **SG-R / SG-rev** | **SG-rev / SG-S** | **Functional category** |
| SA1365 |  | Glycine dehydrogenase (decarboxylating) subunit 2 homolog | - 2.18 | (- 1.49; p = 0.210) |  | Central intermediary metabolism |
| SA2183^a^ |  | Hypothetical protein, similar to nitrate reductase delta | - 2.20 | (- 2.23; p= 0.246) |  | Central intermediary metabolism: Nitrogen metabolism |
| SA2187^a^ | *nasE* | Assimilatory nitrite reductase | - 2.62 | (- 2.08; p = 0.299) |  | Central intermediary metabolism: Nitrogen metabolism |
| SA2189 ^a^ |  | Hypothetical protein, similar to NirR | - 7.07 | (- 2.90; p = 0.198) |  | Central intermediary metabolism: Nitrogen metabolism |
| SA2312 | *ddh* | D-specific D-2-hydroxyacid dehydrogenase | - 3.60 | (- 2.74; p = 0.062) |  | Central intermediary metabolism: pyruvate metabolism |
| SA2413* |  | Sulfite reductase flavoprotein (NADPH) | (1.78; p = 0.092) | 2.76 |  | Central intermediary metabolism: Sulfur metabolism |
| SACOL2024 | *agrD* | AgrD protein | - 3.28 | - 3.14 |  | Signal transduction |
| **SA1701***^a,b^ | *vraS* | Two-component sensor histidine kinase | 4.58 | 6.86 |  | Signal transduction |
| **SA1700*** | *vraR* | Two-component response regulator | 3.01 | 4.39 |  | Signal transduction |
| SACOL2023 | *agrB* | Accessory gene regulator B | - 3.54 | - 3.67 |  | Signal transduction |
| SACOL2025 | *agrC* | Accessory gene regulator C | - 3.65 | - 3.34 |  | Signal transduction |
| SACOL2026 | *agrA* | Accessory gene regulator A | - 4.62 | (- 2.94; p = 0.075) |  | Signal transduction |
| SA2326 | *ptsG* | PTS system, glucose-specific IIABC component | - 2.54 | (- 1.70; p = 0.082) |  | Signal transduction: PTS |
| **SA0189** | *hsdR* | Type I restriction modification enzyme | - 3.64 | - 2.31 |  | DNA metabolism: Restriction/modification |
| SA0353 | *ssb* | Single-strand DNA-binding protein of phage phi PVL | - 2.28 | - 1.72 |  | DNA metabolism: DNA replication, recombination, and repair |
| **SA0746** | *nuc* | Staphylococcal nuclease | - 4.63 | - 4.69 |  | DNA replication and repair |
| SA1282*^a,b^ | *recU (prfA)* | Recombination protein U homolog | (1.90; p =0.070) | 2.59 |  | DNA metabolism: DNA replication, recombination, and repair |
| SA1929 | *pyrG* | CTP synthase | - 2.67 | - 2.33 |  | Purines, pyrimidines, nucleosides, and nucleotides: Pyrimidine ribonucleotide biosynthesis |
| **SA0022** |  | Hypothetical protein, similar to 5-nucleotidase | - 2.68 | - 2.33 |  | Nucleotide transport and metabolism |
| SA0182 |  | Hypothetical protein, similar to indole-3-pyruvate  decarboxylase | - 3.70 | (- 2.25 ; p = 0.088) |  | Carbohydrate transport and  metabolism/Coenzyme metabolism |
| **SA0325** | *glpT* | Glycerol-3-phosphate transporter | - 2.96 | - 2.22 |  | Carbohydrate transport and metabolism |
| SA2053^a^ |  | Glucose uptake protein homolog | - 2.42 | - 2.53 |  | Carbohydrate transport and metabolism |
| SA1269^a^ |  | Major facilitator transporter | - 3.39 | (- 2.93; p = 0.171) |  | Drug resistance transporters |
| SA0128 | *sodM* | superoxide dismutase | - 5.67 | - 5.43 |  | Cellular processes: Detoxification |
|  |  |  | **Fold change** | | |  |
| **ORF annotation** | **Gene** | **Product of putative function** | **SG-R / SG-S** | **SG-R / SG-rev** | **SG-rev / SG-S** | **Functional category** |
| SAUSA300_1964/ SAV1991 |  | Conserved hypothetical phage protein | 2.74 | 2.37 |  | Mobile and extrachromosomal element functions: Prophage functions |
| **SA0091** | *plc* | 1-phosphatidylinositol phosphodiesterase | - 3.21 | - 2.46 |  | Fatty acid and phospholipid metabolism: Degradation |
| **SA0204** | *acpD* | NAD(P)H dehydrogenase homologue | - 5.44 | - 5.78 |  | Fatty acid and phospholipid metabolism |
| SA0309^a^ | *geh* | glycerol ester hydrolase | - 4.27 | - 4.67 |  | Fatty acid and phospholipid metabolism: Degradation |
| SA1073 | *fabD* | Malonyl CoA-acyl carrier protein transacylase | - 2.16 | (- 1.76; p = 0.16) |  | Fatty acid and phospholipid metabolism: Biosynthesis |
| SA1074 | *fabG* | 3-oxoacyl- reductase | - 2.22 | - 1.94 |  | Fatty acid and phospholipid metabolism: Biosynthesis |
| SA2480***** | *drp35* | DPR35 | 2.99 | 3.22 |  | Beta-lactam resistance |
| SA0007 |  | Conserved hypothetical protein | -2.55 | - 2.29 |  | Unknown |
| **SAS016**^a,b^ |  | Hypothetical protein | 21.13 | 51.87 | - 2.45 | Unknown |
| SAS020 |  | Hypothetical protein, similar to phosphoglycerate mutase | - 1.96 | - 2.13 |  | Unknown |
| SAS028 |  | Hypothetical protein | - 2.02 | - 1.2 | - 1.69 | Unknown |
| **SAS037** |  | Hypothetical protein | 2.42 | 2.12 |  | Unknown |
| SA0175 |  | Conserved hypothetical protein | - 4.24 | -3.57 |  | Unknown |
| SA0181 |  | Hypothetical protein, similar to isochorismatase | - 2.16 | -1.73 |  | Unknown |
| SA0183 | *glcA* | PTS enzyme II, glucose-specific, factor IIA homologue | - 4.08 | -3.27 |  | Unknown  (Amino acid and sugar metabolism) |
| **SA0213** |  | Conserved hypothetical protein | - 2.23 | -2.35 |  | Unknown |
| **SA0228** |  | Hypothetical protein | - 4.66 | -4.24 |  | Unknown |
| SA0231^a^ |  | Hypothetical protein, similar to flavohemoprotein | - 4.52 | (-3.08; p = 0.23) |  | Unknown |
| SA0267 |  | hypothetical protein | - 2.48 | - 1.89 |  | Unknown |
| SA0269 |  | Hypothetical protein | - 6.45 | - 4.42 |  | Unknown |
| SA0271 |  | conserved hypothetical protein | - 2.85 | - 2.91 |  | Unknown |
| SA0395 |  | Hypothetical protein | 3.11 | 2.40 |  | Unknown |
| SA0423^a^ |  | Hypothetical protein, similar to autolysin (N-acetylmuramoyl-L-alanine amidase Sle1 precursor) | - 2.11 | - 2.32 |  | Unknown |
| SA0530 |  | Hypothetical protein, similar to indigoidine systhesis protein | - 4.26 | - 4.42 |  | Unknown |
| SA0543 |  | Conserved hypothetical protein | - 2.04 | - 1.70 |  | Unknown |
| SA0551 |  | Mercuric reductase homologue | - 2.05 | - 1.45 |  | Unknown |
| SA0552 |  | Hypothetical protein | - 2.36 | - 1.50 | - 1.59 | Unknown |
| SAS0630 |  | Putative exported protein | 1.96 | 2.06 |  | Unknown |
|  |  |  | **Fold change** | | |  |
| **ORF annotation** | **Gene** | **Product of putative function** | **SG-R / SG-S** | **SG-R / SG-rev** | **SG-rev / SG-S** | **Functional category** |
| SA0645 |  | Conserved hypothetical protein | - 2.34 | (- 1.97; p = 0.058) |  | Unknown |
| SA0666 |  | 6-pyruvoyl tetrahydrobiopterin synthase homologue | - 2.13 | (- 1.47; p = 0.144) |  | Unknown |
| SA0667 |  | Hypothetical protein | - 2.21 | -1.89 |  | Unknown |
| SA0681 |  | Hypothetical protein, similar to multidrug resistance protein | 1.94 | 2.15 |  | Unknown |
| **SA0755** |  | Hypothetical protein, similar to general stress protein | 1.98 | 2.12 |  | Unknown |
| SA0817 |  | Hypothetical protein, similar to NADH-dependent flavin oxidoreductase | (1.52; p = 0.103) | 2.21 |  | Unknown |
| **SA0824** |  | Conserved hypothetical protein | 4.06 | 4.73 |  | Unknown |
| SA0882 |  | Hypothetical protein, similar to competence transcription factor (ComK) | - 2.02 | (-1.95; p= 0.123) |  | Unknown |
| **SA0914***^a,b^ |  | Hypothetical protein, similar to chitinase B | 3.19 | 3.89 |  | Unknown |
| SA0959 |  | GTP-binding elongation factor homolog | - 2.44 | - 2.04 |  | Unknown |
| SA0966 |  | Conserved hypothetical protein | - 3.05 | - 2.87 |  | Unknown |
| **SA1002** |  | Hypothetical protein | - 1.99 | - 2.09 |  | Unknown |
| **SA1015** |  | Hypothetical protein | - 5.58 | - 5.09 |  | Unknown |
| SA1017 |  | Hypothetical protein | - 2.42 | - 2.78 |  | Unknown |
| SA1020 |  | Conserved hypothetical protein | - 2.16 | - 1.81 |  | Unknown |
| SA1040 |  | Conserved hypothetical protein | - 2.03 | - 1.43 |  | Unknown |
| SA1049^a,b^ |  | Hypothetical protein | 2.20 | 1.64 |  | Unknown |
| SA1152 |  | Hypothetical protein | - 2.96 | - 2.23 |  | Unknown |
| SA1254*^a,b^ |  | Conserved hypothetical protein | 3.90 | 3.73 |  | Unknown |
| **SA1265** |  | Conserved hypothetical protein | - 2.90 | - 2.85 |  | Unknown |
| SA1316 |  | Hypothetical protein | - 1.80 | - 2.01 |  | Unknown |
| SA1472 |  | conserved hypothetical protein | - 1.98 | - 2.15 |  | Unknown |
| **SA1476*** |  | Hypothetical protein | 11.65 | 21.97 | - 1.88 | Unknown |
| **SA1514** |  | Hypothetical protein | - 2.31 | - 2.19 |  | Unknown |
| SA1576 |  | Conserved hypothetical protein | - 2.44 | - 1.73 |  | Unknown |
| **SA1617** |  | Hypothetical protein  similar to latent nuclear antigen | - 2.84 | (-1.99; p= 0.195) |  | Unknown |
| **SA1702***^a,b^ | *yvqF* | Conserved hypothetical protein | 5.71 | 7.45 |  | Unknown |
| **SA1703***^a,b^ |  | Hypothetical protein | 7.19 | 8.61 |  | Unknown |
| SA1705 |  | Conserved hypothetical protein | - 2.04 | (-1.86; p= 0.0503) |  | Unknown |
| **SA1712***^a,b^ |  | Hypothetical protein | 5.44 | 5.37 |  | Unknown |
| SA1738 |  | Hypothetical protein | 2.10 | (2.16; p=0.122) |  | Unknown |
| SA1803 |  | Hypothetical protein | 5.40 | 5.36 |  | Unknown |
|  |  |  | **Fold change** | | |  |
| **ORF annotation** | **Gene** | **Product of putative function** | **SG-R / SG-S** | **SG-R / SG-rev** | **SG-rev / SG-S** | **Functional category** |
| SA1840 |  | Conserved hypothetical protein | - 2.78 | - 2.77 |  | Unknown |
| **SA1898** |  | Hypothetical protein similar to SceD protein | 2.18 | 2.33 |  | Unknown |
| SA1942 |  | Hypothetical protein | -2.21 | (-2.15; p=0.092) |  | Unknown |
| SA2001 |  | Hypothetical protein, similar to oxidoreductase, aldo/keto reductase | 3.69 | 3.70 |  | Unknown |
| SA2062 |  | Hypothetical protein | - 2.69 | -2.09 |  | Unknown |
| **SA2113*** |  | Hypothetical protein | 9.79 | 12.58 |  | Unknown |
| SA2191 |  | Hypothetical protein, similar to NirC | - 2.08 | -1.96 |  | Unknown |
| SA2192 |  | Hypothetical protein | - 3.25 | -3.29 |  | Unknown |
| **SA2221***^a,b^ |  | Hypothetical protein | 6.88 | 7.48 |  | Unknown |
| SA2268^a^ |  | Hypothetical protein | - 13.96 | -13.12 |  | Unknown |
| **SA2297***^a,b^ |  | Hypothetical protein, similar to GTP-pyrophosphokinase | 3.06 | 4.19 |  | Unknown |
| SA2310 |  | Conserved hypothetical protein | - 3.40 | -2.75 |  | Unknown |
| SA2328 |  | Conserved hypothetical protein | - 2.01 | (-1.47; p= 0.316) |  | Unknown |
| SA2329 |  | Conserved hypothetical protein | - 2.04 | -1.89 |  | Unknown |
| **SA2343***^a,b^ |  | Hypothetical protein | 50.92 | 64.19 |  | Unknown |
| **SA2377** |  | Conserved hypothetical protein | - 5.09 | - 5.01 |  | Unknown |
| SA2431 | *isaB* | Immunodominant antigen B | - 1.50 | - 2.38 | 1.60 | Unknown |
| **SA2432** |  | Conserved hypothetical protein | - 4.30 | - 3.74 |  | Unknown |
| SA2479^a^ |  | Conserved hypothetical protein | - 2.05 | - 1.97 |  | Unknown |
| SA2481 ^a,b^ |  | Conserved hypothetical protein | 3.46 | 3.65 |  | Unknown |
| SA2487 |  | Hypothetical protein, similar to RarD protein | - 3.32 | -3.03 |  | Unknown |
| **SAUSA300_0768 / SACOL0850** |  | Conserved hypothetical protein | - 2.16 | -1.77 |  | Unknown |
| SAUSA300_0938 / SACOL1042 |  | Conserved hypothetical protein | - 4.39 | -4.15 |  | Unknown |
| SAUSA300_1210 / SACOL1345 |  | Conserved hypothetical protein | - 3.44 | -2.72 |  | Unknown |
| SAUSA300_1211 / SACOL1346 |  | Conserved hypothetical protein | - 3.70 | -2.91 |  | Unknown |
| SAUSA300_1212/ SACOL1347 |  | Conserved hypothetical protein | - 2.26 | -2.14 |  | Unknown |
| SACOL1999 |  | Hypothetical protein | 2.38 | (1.63; p = 0.086) |  | Unknown |
| SACOL2433 |  | Hypothetical protein | - 2.34 | -1.95 |  | Unknown |
| SACOL2559 |  | Hypothetical protein | - 2.21 | (-2.25; p= 0.0506) |  | Unknown |
