# Supplementary material for: Genetic Pathway in Acquisition and Loss of Vancomycin Resistance in a Methicillin Resistant Staphylococcus aureus (MRSA) Strain of Clonal Type USA300
Source: PLoS Pathog. 2012 Feb 2;8(2):e1002505. doi: 10.1371/journal.ppat.1002505 (PMC3271070; doi:10.1371/journal.ppat.1002505)
Supplement: Table S2 — Primers used in sequencing. (DOC) [file ppat.1002505.s004.doc]

| **Primer** | **Sequence (5’ – 3’)** | **Gene** |
| --- | --- | --- |
| yycHUp | GCGACGGTATCTAATGTGAA | *yycH* |
| yycHLower | TGGTGCAAAAACATGCGTCG |  |
| vraG Up | CGTCTCTCAATTTGGATTCG | *vraG* |
| vraG lower | CCCATTACTATGAAAACCGG |  |
| lspAUp | CGAAATAATGGTGCTGCATG | *lspA* |
| lspAlower | CTTAACCTCCTTCTCCTTTT |  |
| Yvq Up | GGCGACCTACATATTGACTT | *yvqF* |
| Yvq lower | GCGAGTACCGAACCAACAAT |  |
| VraSF3 | CGAAGTTAGAACCACCATTA | *vraS* |
| VraSLP3 | ACCTCGATACGTGTACCTGA |  |
| Inter1Up1 | GGGTGGTGCCTATGGCATTA | Intergenic region |
| Inter1lower1 | GTGCAGTGTATAAATAGACG |  |
