# Supplementary material for: Genetic Pathway in Acquisition and Loss of Vancomycin Resistance in a Methicillin Resistant Staphylococcus aureus (MRSA) Strain of Clonal Type USA300
Source: PLoS Pathog. 2012 Feb 2;8(2):e1002505. doi: 10.1371/journal.ppat.1002505 (PMC3271070; doi:10.1371/journal.ppat.1002505)
Supplement: Table S3 — Primers used in the qRT-PCR studies. (DOC) [file ppat.1002505.s005.doc]

| **Primer** | **Sequence (5’ – 3’)** | **Gene** |
| --- | --- | --- |
| qRT -stpC 1 | CCGCTTTGGAAACTAGAGTAACCT | *stpC* |
| qRT -stpC 2 | ATTTAAGCTGAAACGCGGACA |
| qRT -sspB 1 | AGCGTTCCCAACAACTGCCAT | *sspB* |
| qRT -sspB 2 | ACTCAGGGAAGAAGTCCACAAC |
| qRT -prSA 1 | CATCTAAGCCTTCTTTGTCGCT | *prsA* |
| qRT -prsA 2 | ACAGCAAGGTTTAACAGCCGAT |
| qRT -lctE 1 | CGCTTTGCCCTCAGGACGTT | *lctE* |
| qRT -lctE 2 | CGTTCGATGTTGCGCCACGTA |
| qRT -narK 1 | AGAATCCTCCTAAACCACCCAT | *narK* |
| qRT -narK 2 | CGCATTATTCACTGTAGGCTGT |
| qRT -agrA 1 | GTTTCTCACCGATGCATAGCAG | *agrA* |
| qRT -agrA 2 | TAGCTTTGTCGTCAATCGCCAT |
| qRT -spa 1 | TTGCTCACTGAAGGATCGTCT | *spa* |
| qRT -spa 2 | CTCAAGCACCAAAAGCTGACA |
| qRT –SA1007 1 | CGAAGTCTGGTGAAAACCCTG | *orf* SA1007 |
| qRT –SA1007 2 | AGAGATTCTTGGAACCCGGTA |
| qRT –sarH1 1 | GCTGCGCGTCATCCATATGAA | *sarH1* |
| qRT –sarH 2 | CCACCATAAATACCCTCAAACTGT |
| qRT –ssrA 1 | TATCAGCAAGTACGCGATGTG | *ssrA* |
| qRT –ssrA 2 | CGTGTTGAAGGTTTTGAATCTGG |
| qRT – rot 1 | TGCTCTACTTGCAATCGCATC | *rot* |
| qRT –rot 2 | AACCGTATAAGCGTACGAGAAC |
| qRT- SA1813 1 | ACCCTTCAGACACAGTTACAGG | *orf* SA1813 |
| qRT-SA1813 2 | GGGACATGACCATACGAGACAA |
| qRT- lytM 1 | TCGCATGACCACTAGCTGTCG | *lytM* |
| qRT-lytM 2 | AGCGAGTCAAAGCCAACAGCAT |
| qRT-sgtB 1 | ACCTTGCACATCTCTGTCGCTA | *sgtB* |
| qRT- sgtB 2 | ACACGCGATAATGTGGATGAAC |
| qRT-sirA 1 | ACGCGACAATTAAGTCCGGTTT | *sirA* |
| qRT- sirA 2 | TCAAGGTGCCACTGACGTCGCT |
| qRT-acpD 1 | ATGATGGCACACCTAAGAATGT | *acpD* |
| qRT- acpD 2 | CTGCTGAAGGACCACAAGGTT |
| qRT-sodM 1 | CCACCGCCATTATTACGGACT | *sodM* |
| qRT- sodM 2 | ACGCAACAGTTGAAGGAACAGA |
| qRT-SA2221 | TGCTGGTTAATTTGGCCGTCT | *orf* SA2221 |
| qRT- SA2221 2 | AAACGAGAAATCCCGCCGTTC |
| qRT-orf1 1 | CTTGATTATTGATTTCTCTTGCAC | *orf1* (SA1703) |
| qRT- orf1 2 | ACTACCTTTTAATGCTAGATGA |
| qRT-vraS 1 | ACTAGCACGAGAACTTCACGA | *vraS* |
| qRT -vraS 2 | TGTCTTTTAAACCAAGCGGTCT |
| qRT -pta 1 | AGAAGCAATCATTGATGGCGA | *pta* |
| qRT -pta 2 | ACCTGGCGCTTTTTTCTCAG |
